# Validation of musculoskeletal segmentation model with uncertainty estimation for bone and muscle assessment in hip-to-knee clinical CT images

**DOI:** 10.1038/s41598-024-83793-7

**Published:** 2025-01-02

**Authors:** Mazen Soufi, Yoshito Otake, Makoto Iwasa, Keisuke Uemura, Tomoki Hakotani, Masahiro Hashimoto, Yoshitake Yamada, Minoru Yamada, Yoichi Yokoyama, Masahiro Jinzaki, Suzushi Kusano, Masaki Takao, Seiji Okada, Nobuhiko Sugano, Yoshinobu Sato

**Affiliations:** 1 Division of Information Science, Graduate School of Science and Technology, Nara Institute of Science and Technology, 8916-5 Takayama-cho, Ikoma, Nara 630-0192 Japan; 2 Department of Orthopedic Surgery, Graduate School of Medicine, Osaka University, 2-2 Yamadaoka, Suita, Osaka 565-0871 Japan; 3 Department of Radiology, Keio University School of Medicine, 35 Shinanomachi, Shinjuku-ku, Tokyo, 160-8582 Japan; 4 Hitachi Health Care Center, Hitachi Ltd., 4-3-16 Ose, Hitachi, 307-0076 Japan; 5 Department of Bone and Joint Surgery, Graduate School of Medicine, Ehime University, Shitsukawa, Toon, Ehime 791-0295 Japan; 6 Department of Orthopaedic Medical Engineering, Graduate School of Medicine, Osaka University, 2-2 Yamadaoka, Suita, Osaka 565-0871 Japan

**Keywords:** Biomedical engineering, Skeletal muscle

## Abstract

Deep learning-based image segmentation has allowed for the fully automated, accurate, and rapid analysis of musculoskeletal (MSK) structures from medical images. However, current approaches were either applied only to 2D cross-sectional images, addressed few structures, or were validated on small datasets, which limit the application in large-scale databases. This study aimed to validate an improved deep learning model for volumetric MSK segmentation of the hip and thigh with uncertainty estimation from clinical computed tomography (CT) images. Databases of CT images from multiple manufacturers/scanners, disease status, and patient positioning were used. The segmentation accuracy, and accuracy in estimating the structures volume and density, i.e., mean HU, were evaluated. An approach for segmentation failure detection based on predictive uncertainty was also investigated. The model has improved all segmentation accuracy and structure volume/density evaluation metrics compared to a shallower baseline model with a smaller training database (N = 20). The predictive uncertainty yielded large areas under the receiver operating characteristic (AUROC) curves (AUROCs ≥ .95) in detecting inaccurate and failed segmentations. Furthermore, the study has shown an impact of the disease severity status on the model’s predictive uncertainties when applied to a large-scale database. The high segmentation and muscle volume/density estimation accuracy and the high accuracy in failure detection based on the predictive uncertainty exhibited the model’s reliability for analyzing individual MSK structures in large-scale CT databases.

## Introduction

The advent of deep learning (DL)-based image segmentation has allowed for the fully automated, accurate, and rapid analysis of MSK structures from medical images^1–10^. These models assist in extracting the structure’s shape and estimating diagnostic image biomarkers, such as volume and muscle density, for assessing muscle atrophy and fatty degeneration^11,12^. These biomarkers can be used for the diagnosis of muscle pathologies, such as muscular dystrophy^13^, cachexia^14^, and sarcopenia^15^. The models were tested on the rotator cuff^8^, chest^3^, hip and thigh^7^, and abdominal muscles^1,2,4,9,10,16,17^. However, multiple issues exist in those studies that limit the reliable application in large-scale databases.

- Several studies addressed the segmentation of the muscles in only a single or a few two-dimensional (2D) CT slices^1,18,19^, which do not reflect the three-dimensional (3D) properties of the muscles and depend on the subjective selection of the slices^20^.
- The 3D muscle segmentation has also been attempted^8,10,21^; however, only a few muscles were addressed. A recent study^22^ addressed the 3D segmentation of 27 hip and thigh muscles in CT images; however, the model was tested on a small database consisting of 12 cases, and the average accuracy was lower than that reported in a previous study targeting similar muscles^7^.
- Current muscle segmentation approaches assess the model’s accuracy only in cross-sectional area or volume estimation. However, muscle density, which can be quantified based on the mean Hounsfield units (HU) in the CT image^23^, has shown higher correlations with muscle strength and functions^11,24^. This necessitates the accuracy assessment of muscle density estimation, as well.
- Even though some studies attempted the analysis of large-scale databases^21,25^, no rigid criteria were applied for segmentation failure detection. In other words, it is not clear how to determine whether the automatic predictions can be safely adopted, possibly corrected with moderate efforts, or better excluded for a reliable downstream analysis.

Our group has developed a segmentation tool, i.e., Bayesian UNet with Monte-Carlo dropout sampling (MCDS), that outputs the model’s uncertainty, a.k.a *predictive uncertainty* in addition to the target segmentations using multiple predictions from random dropouts of the model weights (i.e. dropout samples) at the inference time^7^. The model was validated on a database of 20 cases of hip osteoarthritis (hip OA) patients. It has shown high accuracy in segmenting 19 hip and thigh muscles as well as the possibility of predicting the segmentation accuracy in *unannotated* CT images based on the predictive uncertainty. In the future, we want to leverage this tool to segment large-scale databases of CT images collected from many health centers^26^, and analyze the impact of the demographic and disease factors in the Japanese population. These databases include large variations from the training data, such as manufacturer/scanner, imaging conditions, and disease variations, which may lead to segmentation failure due to the domain shift problem^27–30^. Furthermore, even though the number of dropout samples was found to affect the accuracy of MCDS-based approaches^31^, the impact of this parameter on the MSK segmentation was not investigated. Fitzpatrick et al. reported the automated volumetric and demographic analysis of the iliopsoas muscle segmented from magnetic resonance (MR) images of 5,000 subjects^25^. Their database was collected from the UK Biobank database^32^, which, in contrast to ours, has a unified imaging scanner and protocol that mitigates the domain shift problem. The predictive uncertainty was also addressed in previous studies to predict the segmentation accuracy in *unannotated* images^7,15,33–35^; however, the analysis was limited to small databases, and no quantitative criteria were applied for the detection of the segmentation failures for the down-stream analysis.

In this study, we report the preparations conducted to employ the model for muscle segmentation in the large-scale database. In particular, a larger fully annotated database consisting of 50 cases of hip OA patients acquired by two CT scanners has been prepared. In addition, the model’s capacity has been increased to account for the enlarged training database. The major contributions of this work are as follows:

- Investigating the segmentation accuracy and volume/intensity prediction in 22 MSK structures from four databases of CT images acquired from multiple manufacturers/scanners with various disease conditions, and patient positioning, i.e., standing and supine positions.
- Assessing the accuracy of the predictive uncertainty as a predictor of the segmentation accuracy under various imaging conditions and disease variations and suggesting quantitative criteria for detecting segmentation failures.
- Showcasing the capability of the predictive uncertainty and suggested criteria in detecting segmentation failures at a large database of > 2,500 volumetric CT images of hip OA patients, and investigating the impact of the disease status on the predictive uncertainty.

## Materials and methods

### CT images and annotations

In this study, databases of CT images from multiple manufacturers/scanners, disease status, and patient positioning were used. Table 1 summarizes the characteristics of the databases (DBs) used in this study. DB#1 included pre-operational images from 50 unilateral hip OA patients (mean age: 61.4 ± 13.0 yrs, min: 30 yrs, max: 86 yrs; 44 females, 6 males) acquired by two scanners from different generations by the same manufacturer (HiSpeed ”old” (N=20) and Optima CT660 ”new” (N=30), GE Healthcare, Milwaukee, WI). The images were resampled so the slice interval became 1.0 mm throughout the entire volume. The disease severity was assessed using Crowe^36^ and Kellgren and Lawrence (KL)^37^ grading, in which higher grades indicate higher disease severity. The affected sides were those with KL,Crowe>1. This database was used for the internal validation of the model accuracy, for investigating the impact of the training and inference settings, and for disease status.

**Table 1:** CT image characteristics.

Database	Inst.	Diagnosis	Patient positioning	No. of cases	Modality	Matrix size	In-plane resolution [mm]	Slice interval [mm]
Internal training and testing (fivefold cross-validation)								
DB#1	Osaka Univ Hosp.	Unilateral hip OA	Supine	20	HiSpeed, GE	512^2^	0.703^2^ − 0.742^2^	1.0–6.0*
				30	Optima CT660, GE	512^2^	0.703^2^ − 0.820^2^	1.25
External validation (small-scale, with ground-truth labels of the GMED muscle)								
DB#2	TCIA	Soft tissue sarcoma	Supine	18	Discovery ST, GE	512^2^	0.977^2^	3.75
DB#3	Hitachi Medical Care Center	Colorectal cancer	Supine	10	Supria, Hitachi	512^2^	0.685^2^	0.63
DB#4	Keio Univ Hosp.	Normal	Supine	20	Aquilion ONE, Canon Medical Systems	512^2^	0.683^2^	0.5
			Standing	20	prototype TSX-401R, Canon Medical Systems	512^2^	0.683^2^	0.5
Large-scale predictive uncertainty analysis (without ground-truth labels)								
DB#5	Osaka Univ Hosp.	Uni/ bilateral hip OA	Supine	460	HiSpeed, GE	512^2^	0.703^2^ − 0.742^2^	1.0–6.0 ∗
				2119	Optima CT660, GE	512^2^	0.703^2^ − 0.820^2^	0.675- 3.75

*pelvis and proximal femur: 2.0 mm, femoral shaft region: 6.0 mm, distal femur region: 1.0 mm.

The three databases DB#2-4 were for subjects without hip OA from institutions different from that of DB#1, and were used for external validation of the model accuracy. DB#2 was collected from a public database^38^, including 18 cases (age anonymized; 13 females, 5 males) with soft tissue sarcoma acquired by a scanner from the same manufacturer as DB#1 but a different model (Discovery ST, GE Healthcare, Milwaukee, WI). DB#3 included images for 10 subjects (mean age: 50.1 ±7.6 yrs, min: 41 yrs, max: 64 yrs; 10 males) who were scanned for the diagnosis of colorectal cancer using a scanner from a different manufacturer (Supria, Hitachi Medical, Tokyo, Japan). DB#4 included images of 20 healthy volunteers (mean age: 65.1 ± 6.3 yrs, min: 55 yrs, max: 76). The images were acquired for the volunteers in the supine and standing positions. The supine images were acquired with a 320-row detector CT scanner (Aquilion ONE, Canon Medical Systems Corporation, Otawara, Japan), while the standing images were acquired with an upright 320-row detector CT (prototype TSX-401R, Canon Medical Systems Corporation, Otawara, Japan)^39^.

DB#5 included pre-operational images for uni/bilateral hip OA patients collected from the same institution as DB#1. This database was used to showcase the usability of the predictive uncertainty in failure detection and the impact of disease status in a large-scale setting. The original database contained 9,260 CT images, acquired with hip-to-knee and whole lower limb imaging protocols for total hip arthroplasty (THA) surgery. Digitally reconstructed radiographs (DRRs) were constructed from each CT volume. The DRR was used to visually confirm the presence of metal implants from hip-to-knee, and truncate the original volume below the knee level if the image covered the whole limb. The process ended up with 2,579 CT volumes (mean age: 61.8 ± 15.2 yrs, min: 13 yrs, max: 98 yrs; 2,062 females, 497 males). The affected and unaffected sides in each CT image were assigned based on an automatic grading model^40^. Particularly, a cubic region of interest (ROI) centered at the hip center on each side was extracted using a CNN-based landmark detection tool. A DRR was constructed for each side, and was input to a 7-class disease severity classification model with a vision transformer (ViT) architecture^41^. A pre-trained model with a classification accuracy of .962 was used to predict the disease severity, and the sides with KL,Crowe>1 were considered affected, and unaffected otherwise.

**Fig. 1:**
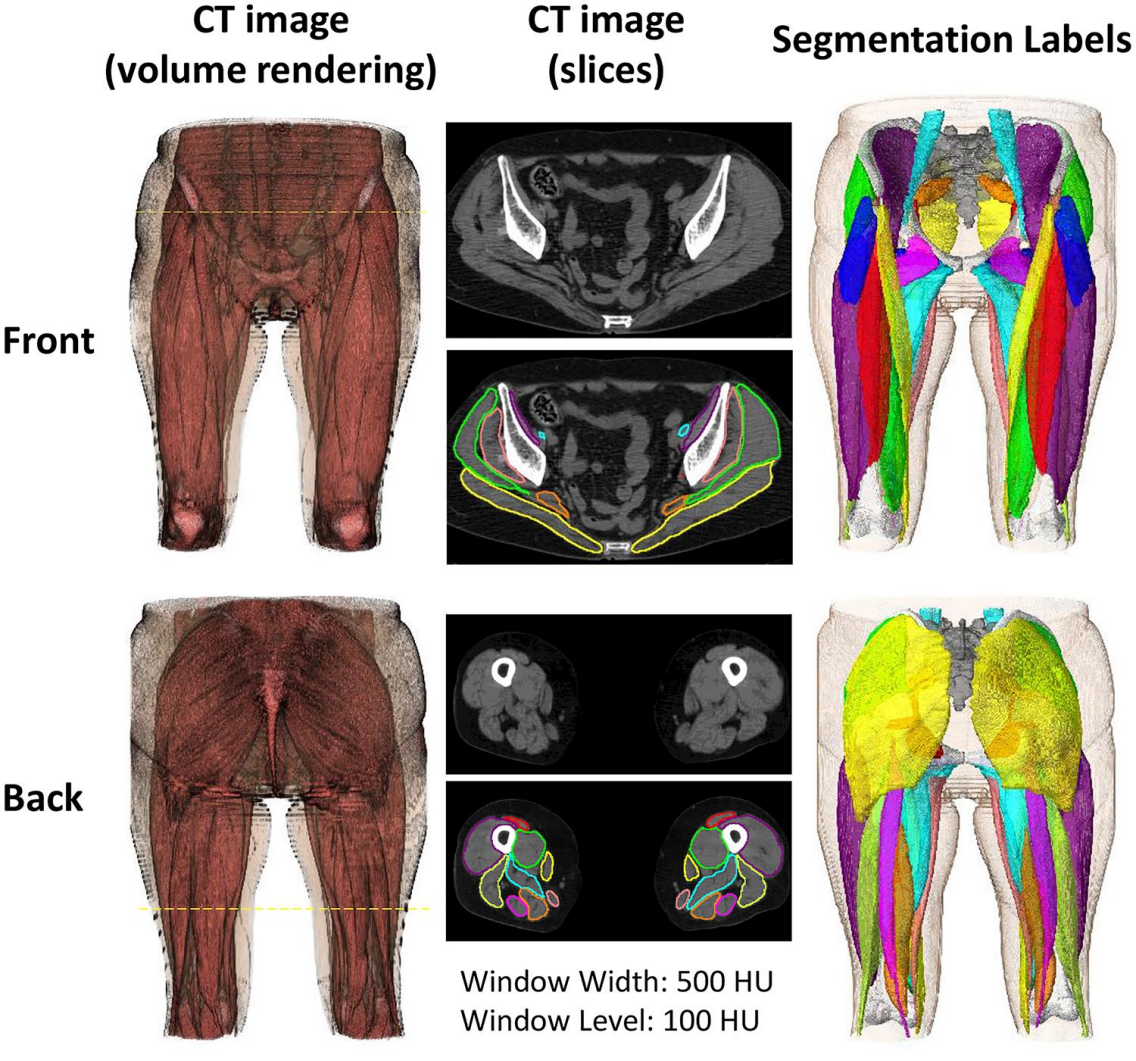
Segmentation labels of the bones and muscles

Figure 1 shows an example of the target structures including 19 muscles and three bones, whereas Table 2 lists the structures names, abbreviations used in text, and the visualization colors. A collaborative group consisting of a health science researcher with a medical physics background, computer science researchers, and orthopedic surgeons specializing in musculoskeletal imaging created and validated the ground-truth (GT) labels of all the target structures in DB#1 and the GMED muscle in DBs#2-4. The annotations of the 50 cases in DB#1 passed through multiple annotation and validation cycles. The annotations were first created using a pre-trained model^7^, and the automated segmentations were corrected using 3D Slicer^42^.

**Table 2:**
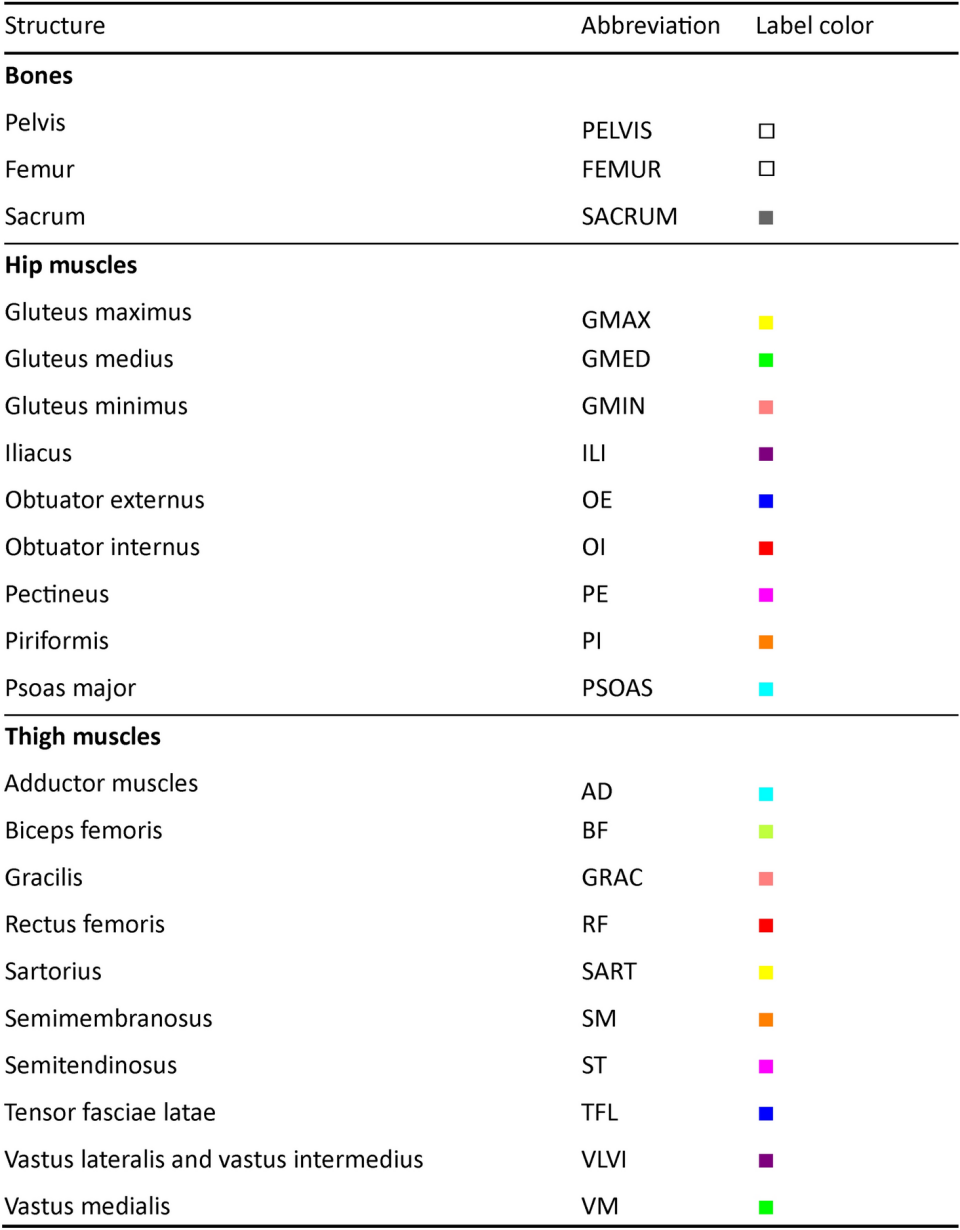
Target structures.

### Overall scheme

**Fig. 2:**
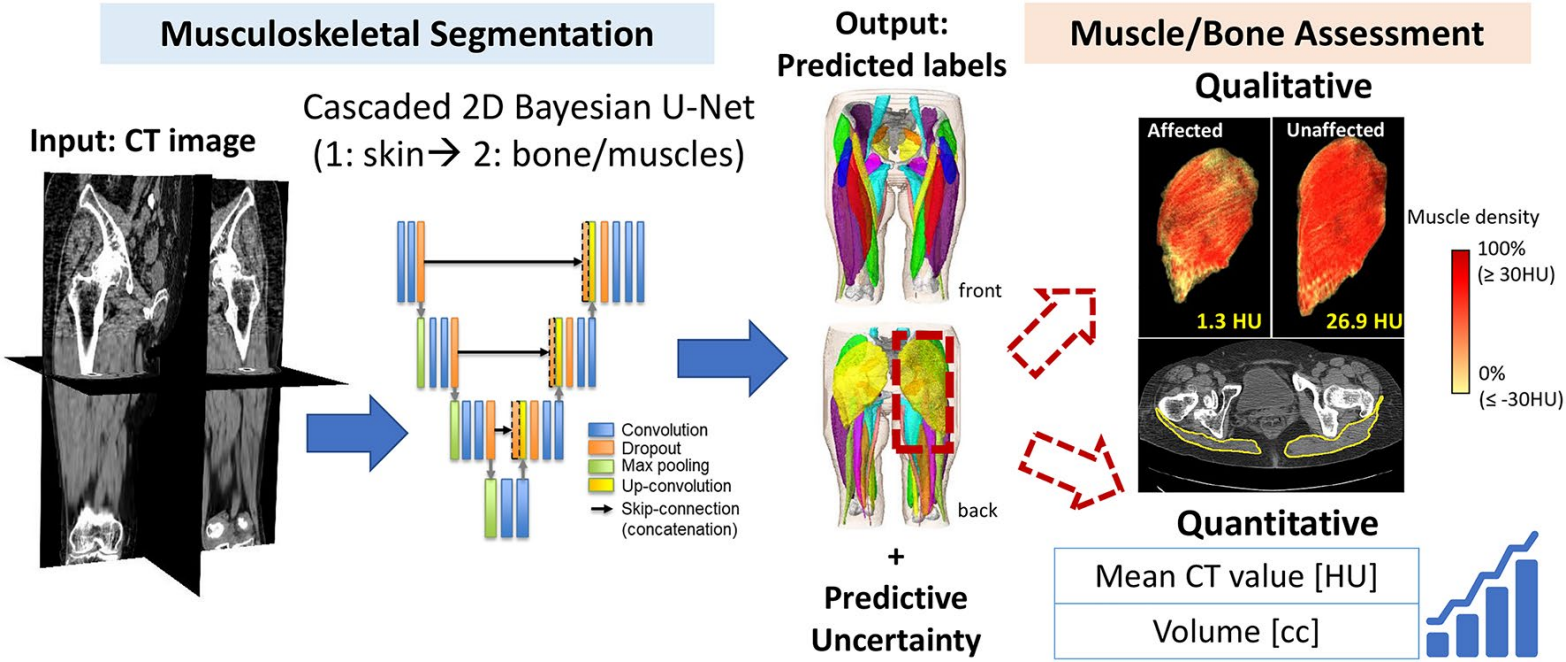
Overall scheme for validation of musculoskeletal segmentation model for automated assessment of bones and muscles in CT images with uncertainty estimation

Figure 2 shows the overall scheme of validating the segmentation model for the automated assessment of bones and muscles in CT images. The CT image was input to the model, where each axial slice was processed to segment the bones and muscles. Each bone and muscle were extracted from the concatenated volume of all slices for qualitative, i.e., muscle density visualization, and quantitative, i.e., volume and mean HU assessments. Besides the bone/muscle labels, the structure-wise predictive uncertainty was computed based on MCDS.^7,31^ The databases and proposed scheme were used to tackle the following research questions:

- DB#1: What is the model’s performance (i.e., segmentation and muscle/bone assessment accuracy) under variations the training settings (i.e., number of UNet encoder/decoder layers and number of training cases), and inference settings (number of dropout samples and estimation method of predictive uncertainty)?
- DB#2–4: How would the model performance change if applied to external databases of CT images from multiple manufacturers/scanners, with various disease conditions and patient positioning (standing and supine positions)?
- DB#5: Can the predictive uncertainty be used for segmentation failure detection in large-scale *unannotated* databases?
- DB#1,5: What is the impact of the disease status on the model performance and predictive uncertainty?

**Fig. 3:**
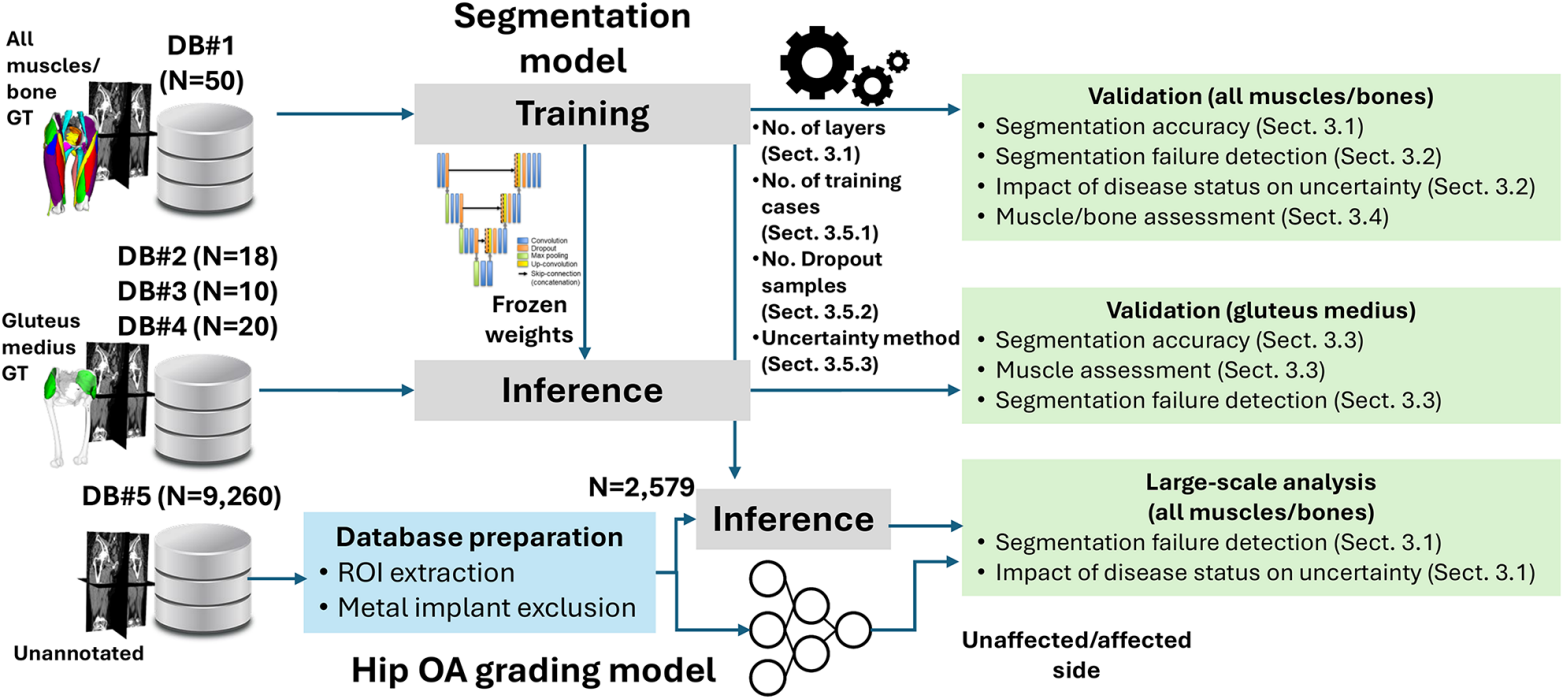
Summary of the research questions tackled in the study with the corresponding databases and methodologies used in the experiments. Sect: section numbers in the paper, ROI: region-of-interest, Hip OA: hip osteoarthritis, GT: ground-truth (annotation), DB: database, N: number of cases

Figure 3 summarizes the research questions in this study with the corresponding databases.

### Image segmentation

In this study, a cascaded 2D Bayesian U-Net model, which outputs the predicted structure labels with pixel-wise predictive uncertainty maps, was used^7^. The baseline model architecture consisted of an encoder and decoder composed of multiple down/upsampling layers (hereinafter called *layers* for simplicity). Each encoder layer consisted of two basic blocks each consisting of padding-convolution-activation blocks, followed by a dropout block. The decoder layer consisted of an upconvolution block, whose output was concatenated with the corresponding encoder layer’s output, followed by a basic block. The model’s output feature map was input to a softmax layer to obtain the voxel-wise class probabilities. Two modifications were made to the baseline architecture: 1) increasing the depth of the model (i.e., using six encoder/five decoder layers instead of five encoder/four decoder layers), and 2) adding a batch normalization layer^43^ to the basic convolutional blocks, which stabilizes the training of large neural network models and improves the overall performance^33^. Similar to^7^, at the inference time, the mean and the variance of 10 MC dropout samples were used to obtain the output label and voxel-wise uncertainty map, respectively. The structure-wise predictive uncertainty was computed as the average of the voxel-wise uncertainty map within the segmented label.

To investigate the impact of the modified model and larger annotated database, including 50 cases, the performance was compared with the baseline model consisting of five encoder/four decoder layers and validated on 20 or 50 cases. For simplicity, the aforementioned models were termed (*5layers,20*), (*5layers,50*), and (*6layers,50*). The parameters were ∼10 M and ∼44 M for the *5layers* and *6layers* models, respectively.

### Muscle/bone assessment

The labels predicted by the segmentation model were used to assess each structure’s volume and muscle density. The volume was computed as a multiplication of the number of voxels by the size of each voxel in centimeter cubes (cc) normalized by the subject’s height. The muscle density was computed as the mean of the intensity or CT values in HU within the segmented label. Structures on the right and left sides of the patient’s body were assessed separately based on a postprocessing using connected component analysis (CCA). An additional watershed algorithm and CCA were used to separate the right and left sides when connected (e.g., the connection of right and left hemi-pelvises at the pubic symphysis).

A transfer function was used to comprehensively convert the HU values into scalar muscle density to visualize lean muscle and intramuscular fat. HU values less than -30 HU were considered fat, values within the range [_−_30*,*30] were considered muscle/fat composite, and values larger than 30 HU were considered lean muscle^23^. Color and opacity transfer functions were used to visualize the transformed image (see Fig. 2, right).

### Evaluation metrics

The segmentation accuracy, and accuracy in estimating the structures volume and density, i.e., mean HU, were evaluated. The segmentation accuracy was evaluated using the Dice coefficient (DC) and average symmetric surface distance (ASD). DC assesses the overlap between the GT and predicted labels. ASD assesses the surface distance, i.e., surface error, to assess the presence of small yet distant false positive structures. The predicted volume and mean HU accuracy were evaluated using the absolute difference between the quantities measured at the GT and predicted labels. The volume error (average volume error [AVE]) was computed as a percentage relative to the GT volume. The intensity error (average intensity error [AIE]) was reported as the average of absolute differences between the mean HUs of the GT and predicted labels.

The accuracy of the predictive uncertainty for detecting inaccurate (correctable with moderate human effort) or failed (correctable with notable efforts) segmentations was investigated. For each structure, a threshold based on the standard deviation of DC was determined to consider the segmentation inaccurate or failed. To make the threshold setting more statistically robust against outliers, the median absolute deviation (MAD) was used. Particularly, a threshold of *Median*_*DC*_+1*.*4826∗k*MAD_*DC*_ was used, where *k* was set as -2 or -3 for inaccurate or failed segmentations, respectively. The area under the receiver operating characteristic (AUROC) curves of the predictive uncertainty based on the DC threshold was used to assess the detection accuracy. The AUROCs of the predictive uncertainty from both *5layers,20* and *6layers,50* were computed. Linear regression lines were computed between DC (dependent) and the predictive uncertainty (independent) for each structure and the averages of all structures combined.

### Statistical analysis

The concordance correlation coefficient (*CCC*)^44^ was used to assess the agreement between the GT and predicted volume and mean HU. The Pearson correlation coefficient (*ρ*) assessed the linear relationship between the predictive uncertainty and DC. To investigate the statistical significance of the differences between paired measurements, the Shapiro test was first used to assess the normality of the different distributions. Student’s t-test was used when normality was found. Otherwise, the Wilcoxon signed-rank test was used. A probability of *p* = 0.05 was considered significant in all tests. Bonferroni correction was used when multiple comparisons between the models or databases were made.

### Implementation details

The proposed approach was developed and validated in Python and Keras^45,46^. The segmentation models (*5layers,20*), (*5layers,50*), and (*6layers,50*) were trained and validated on DB#1 based on 5-fold cross-validation. For a matched comparison among the models, the remaining 30 out of the 50 cases used in training the (*5layers,20*) model were used in the inference phase. Models with 5 and 6 layers were retrained on all the images in DB#1 and were used to predict the labels in DBs#2-5. The quantitative validation of DBs#2-4 was limited to the GMED muscle, whereas the average of the predictive uncertainty in all structures was used in DB#5. The predictive uncertainty thresholds used for failure detection in DB#5 were derived based on the linear regression lines computed in DB#1.

The segmentation model training and inference were performed on a Linux-based cluster of servers with graphical processing units (GPUs; Nvidia Corporation, Santa Clara, CA, USA). Similar to the previous study^7^, the (*5layers,20*) model was trained for 150k iterations with a batch size of 3, whereas the models *5layers,50* and *6layers,50* were trained for 200k iterations due to the increased training data and model capacity. The inference time per volume (approximately 500 CT slices) was approximately 3 minutes.

## Results

### Segmentation accuracy and predictive uncertainty

**Fig. 4:**
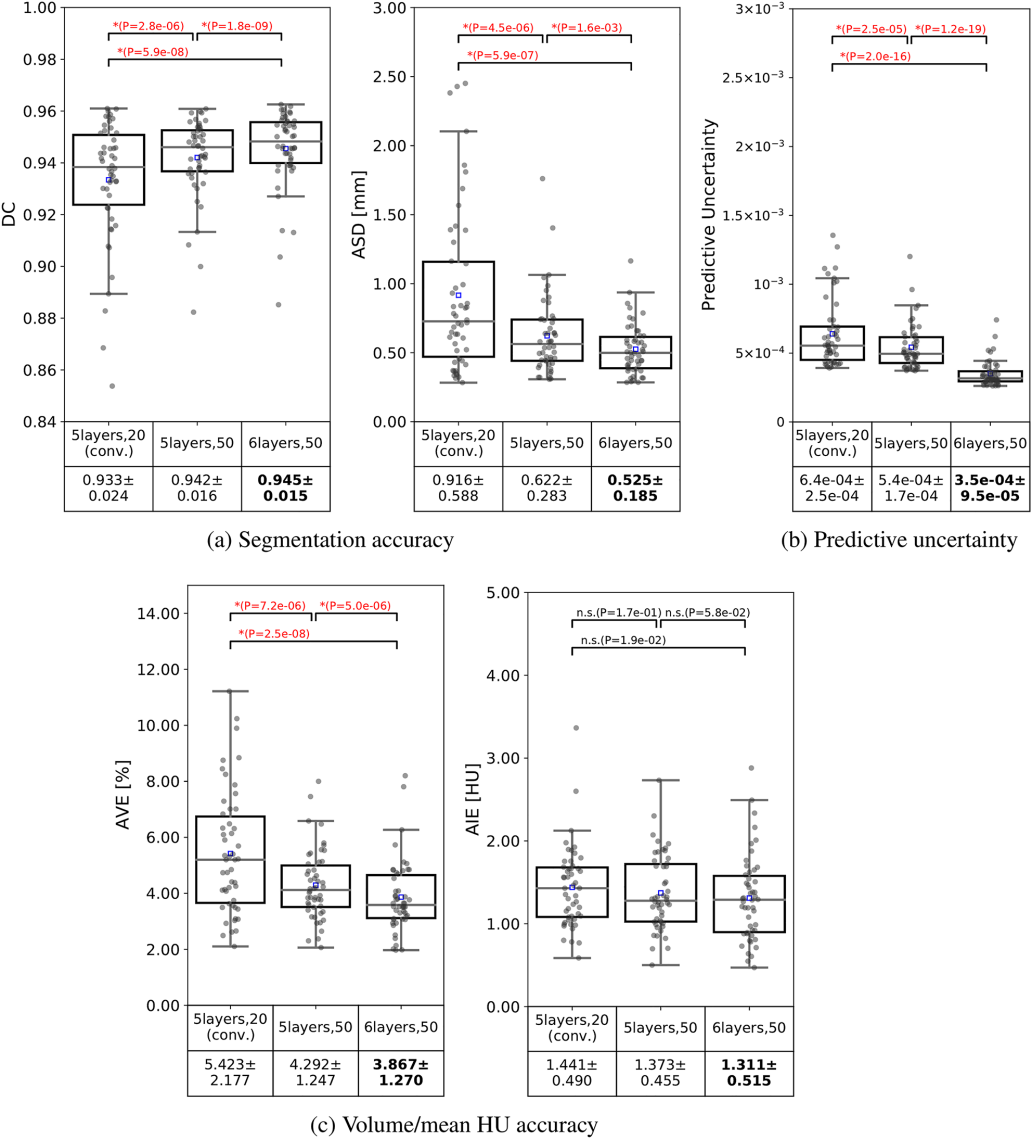
Distributions of the segmentation accuracy (**a**), predictive uncertainty (**b**), and volume/mean HU accuracy (**c**) of the bones and muscles (averaged on all structures) by each model applied to DB#1 (N = 50). Horizontal lines in the boxes represent the medians, while blue boxes represent the means. Detailed values are depicted in Supplementary Figs. A.1-A.5. DC: Dice coefficient, ASD: average symmetric surface distance, AVE: average volume error, AIE: average intensity error, n.s.: not significant, *: *p* < 0.017, Student’s t-test or Wilcoxon signed rank sum test with Bonferroni correction

The improved model *6layers,50* has shown overall improvements with respect to all evaluation metrics. Figure 4 shows the segmentation accuracy, predictive uncertainty, and volume/mean HU accuracy of the three models. Each point represents the average metric value of all structures in a single subject. The accuracy of the *6layers,50* model was significantly higher than that of the *5layers,20* in terms of all metrics. The average DC of the *6layers,50* model was .945±.015, with an average increase of 1.2% at all structures compared with *5layers,20* (*p*<.017). An average improvement of approximately 0.4 mm was observed in ASD (*p*<.017). The improvement by *6layers,50* model was statistically significant in most MSK structures, as shown in Supplementary Figs. A.1 (DC), A.2 (ASD).

The box plots in Fig. 4(b) show that the uncertainty proportionally decreased with the improved segmentation accuracy in Fig. 4(a) regarding the number of training cases and model depth. Scatter plots of DC versus the predictive uncertainty for each model are depicted in Supplementary Fig. A.6(a). Linear relationships with strong correlations were obtained between the segmentation accuracy and the predictive uncertainty by all the models. A strong correlation of *ρ*=-.79 was obtained in the *6layers,50* model. This emphasizes the usability of the predictive uncertainty as a predictor of the segmentation accuracy, which supports the findings by the previous studies^7,33^.

**Fig. 5:**
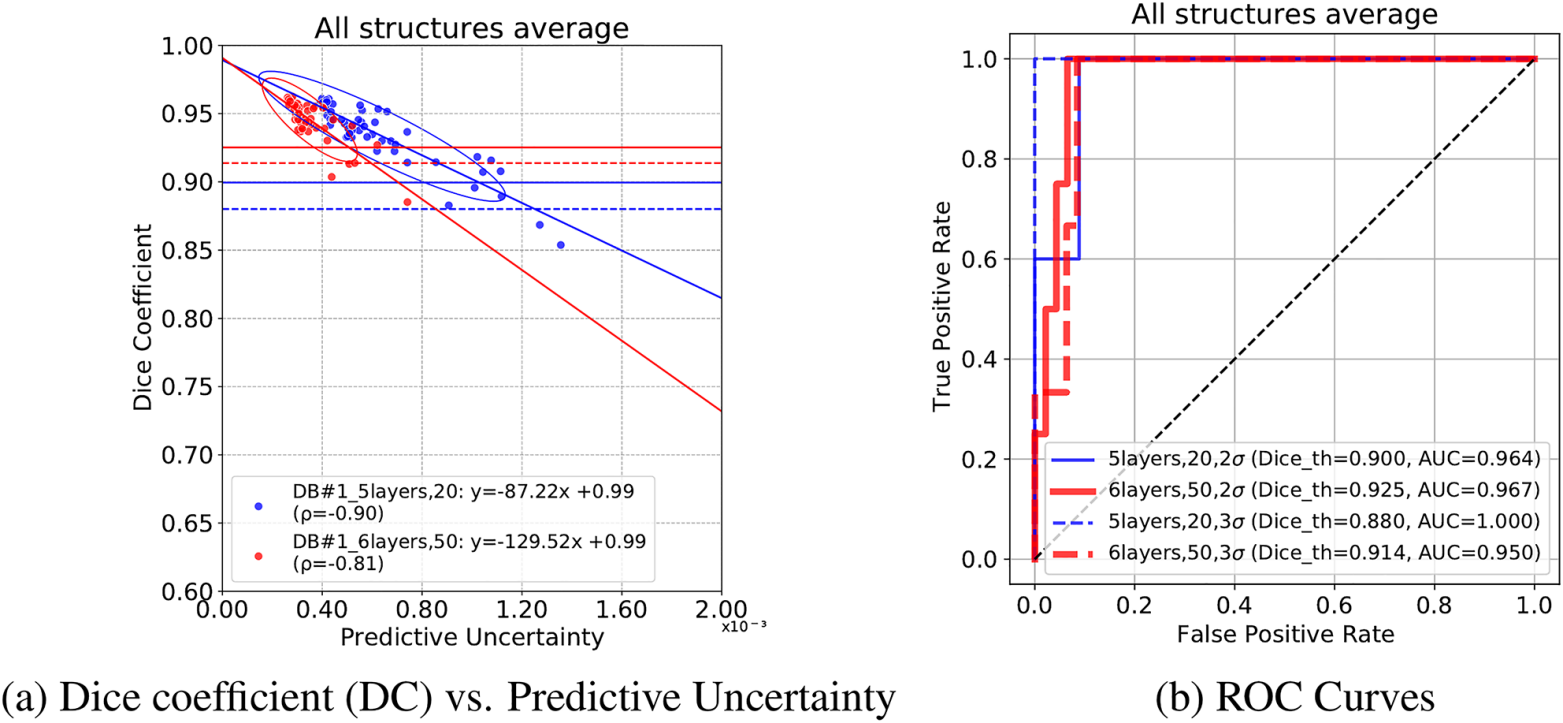
Receiver operating characteristic (ROC) curves of the inaccurate and failed segmentation detection in DB#1 (N = 50) using the predictive uncertainty. Thresholds were determined based on the median absolute deviations (σ) of the DC

Figure 5 shows the relationship between the average DC and average predictive uncertainty of all structures in each patient in DB#1 and the corresponding ROC curve for failure detection. The predictive uncertainty of both models (*5layers,20* and *6layers,50*) yielded high AUROCs (≥.95) in detecting inaccurate and failed segmentations. Table 3 shows the AUROCs of each structure. The median AUROCs of all structures by *6layers,50* for detecting inaccurate and failed segmentations were .979 and .959, respectively. The OE and OI muscles had the lowest accuracy. Supplementary Fig. A.9 shows the detailed results of each structure. Supplementary Figure A.11 shows scatter plots of the predictive uncertainty by the two models in DB#5. Based on the thresholds computed in DB#1 (Fig. 5), three representative cases were visualized. The improved segmentation by the *6layers,50* model can be observed in the three cases and the scatter plot. The representative case of the failed segmentation exhibits unusual positioning of the hip, possibly due to the patient’s discomfort as a result of the disease.

**Table 3:** Accuracy (area under receiver operator curve (AUROC)) of the segmentation failure detection based on the predictive uncertainty and segmentation accuracy (Dice coefficient; DC) of all structures DB#1 (N = 50). σ indicates the threshold computed based on the median absolute deviation of DC and used for the detection of inaccurate (-2σ) and failed (-3σ) segmentations. ”All structures average” indicates using the average of predictive uncertainties of all structures for failure detection. The structures’ abbreviations are listed in Table 2.

		AUROC↑			
Group	Structure	5layers,20		6layers,50	
		-2σ	-3σ	-2σ	-3σ
Hip muscles	GMAX	0.935	0.970	0.874	0.958
	GMED	0.989	0.990	1.000	0.990
	GMIN	0.864	0.802	0.959	0.959
	ILI	0.943	–	0.865	0.837
	OE	0.696	0.592	0.534	0.413
	OI	0.552	–	0.693	–
	PE	0.962	0.990	0.965	0.980
	PI	0.987	1.000	1.000	1.000
	PSOAS	0.991	0.979	0.963	0.916
Thigh muscles	AD	0.936	1.000	0.894	0.990
	BF	0.977	0.967	0.898	0.898
	GRAC	1.000	1.000	0.913	0.996
	RF	0.981	0.996	0.965	0.970
	SART	0.973	0.996	0.876	0.978
	SM	0.924	0.969	0.938	1.000
	ST	0.875	0.867	0.894	1.000
	TFL	0.964	0.908	0.819	0.682
	VLVI	0.920	0.911	0.951	0.940
	VM	0.896	0.984	0.853	0.986
Bones	PELVIS	0.917	0.939	0.891	0.765
	FEMUR	0.995	0.995	0.904	0.948
	SACRUM	0.920	0.958	0.750	0.791
All structures average		0.964	1.000	0.967	0.950
Median		0.943	0.979	0.898	0.965

### Relationship between segmentation accuracy/predictive uncertainty and disease stage

**Fig. 6:**
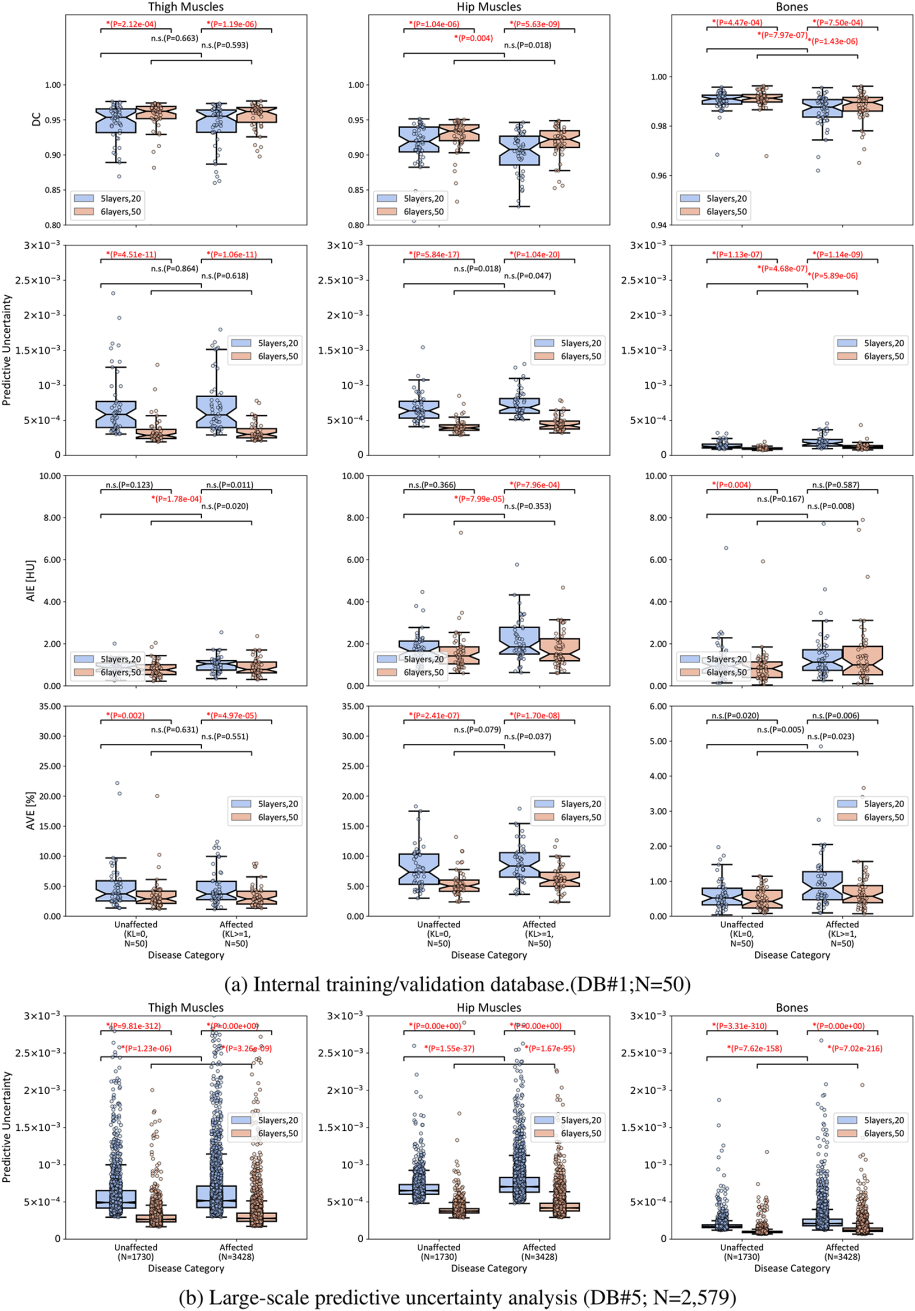
Distributions of the accuracy evaluation metrics and predictive uncertainty of the three MSK structure groups, i.e., thigh (left) and hip (middle) muscles and bones (right), in terms of the disease status of body sides in hip OA patients in internal validation DB#1 (a) and large-scale predictive uncertainty analysis in DB#5) (b). N: number of cases. n.s.: not significant, *: *p* < 0.004. (Based on Shapiro’s normality test, the hypothesis test was performed using either the Wilcoxon signed-rank test or the Student’s t-test. Bonferroni correction was used for the multiple comparisons.)

Figure 6 shows the distributions of the evaluation metrics and predictive uncertainty at the internal DB#1 (a) and predictive uncertainty in the large-scale database DB#5 (b) in terms of the hip OA disease status (unaffected vs. affected) in each body side. The model *6layers,50* showed statistically significant improvement (*p*<.01) in all the structure groups in DB#1. The proportional relationship between the accuracy and predictive uncertainty can also be observed in all structure groups, where smaller uncertainty was accompanied by increasing accuracy. The unaffected sides significantly showed higher accuracy in the bones than the affected ones in DB#1. All groups had a similar tendency in DB#5, where the affected sides had higher predictive uncertainty. In addition to the sensitivity to the variations in the positioning, as shown in Fig. A.11, this shows a possible impact of the disease status on the performance of the segmentation model in large-scale databases.

### Validation on a multi-manufacturer/scanner database

**Fig. 7:**
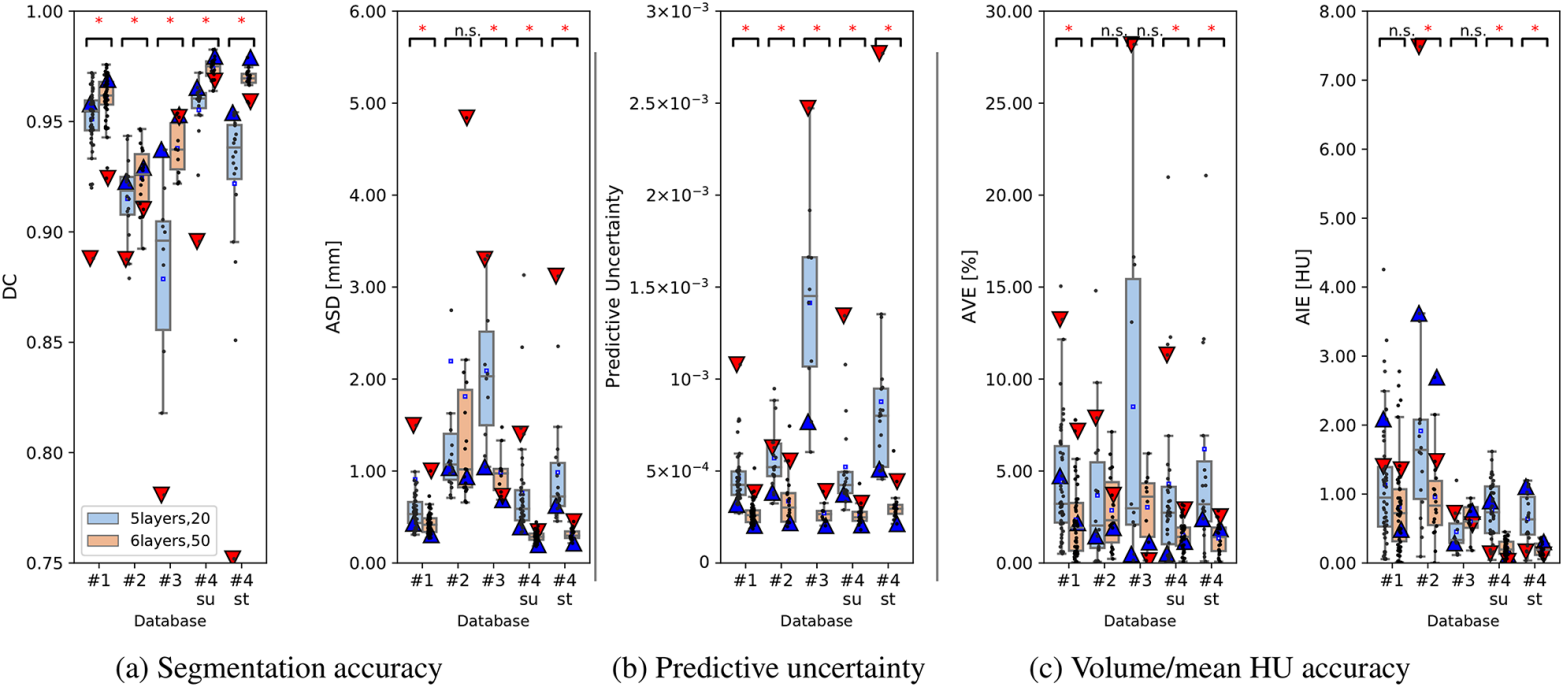
Comparison between segmentation model accuracy (a, c) and predictive uncertainty (b) of the GMED muscle in the multi-manufacturer/scanner databases DB#1(N = 50), DB2(N = 18), DB#3(N = 10) and DB#4(N = 20). DC: Dice coefficient, ASD: Average symmetric surface distance, AVE: Average volume error, AIE: Average intensity error, su: supine, st: standing, n.s.: not significant, *: *p* < 0.01. (Based on Shapiro’s normality test, the hypothesis tests were performed using either the Wilcoxon signed-rank test or the Student’s t-test with Bonferroni correction). The triangles indicate the cases corresponding to the 5th (blue filled triangle) and 95th (red filled upside down triangle) quantiles of the predictive uncertainty visualized in A.7 and A.8.

Figure 7 shows the evaluation metrics and predictive uncertainty of GMED muscle segmented at the databases DB#1-4 from multiple manufacturers/scanners and disease variations (see Table. 1). Representative cases (5^th^ (blue filled triangle) and 95^th^ (red filled upside down triangle) quantiles of the predictive uncertainty visualized in Supplementary Figs.A.7 and A.8) are depicted. Statistically significant improvements in the DC, ASD, and AIE were observed in the four databases using the model *6layers,50*. The predictive uncertainty was obviously related to the accuracy metrics, where low uncertainty cases mostly had high accuracy metric values and vice versa.

Table 4 summarizes the means and SDs of the evaluation metrics in the four databases. Using the *6layers,50* model, the segmentation accuracy at a predictive uncertainty of 5 × 10^−4^ was > *0.9*0 (DC) and approximately less than 2.00 mm (ASD) in all the databases. Notably, a sub-HU accuracy was obtained in predicting the mean HU in the four databases. Overall improvements were observed in all the muscles by the *6layers,50* model (see Supplementary Fig. A.7). At the GMED muscle, the *5layers,20* model failed to capture the boundaries with the GMAX muscle, which reduced its segmentation accuracy, whereas the errors were less in the *6layers,50* model results. The PSOAS muscle’s lower part was undersegmented in multiple instances by the *5layers,20* model. Noteworthy, only a slight degradation in accuracy was observed at standing compared with the supine positioning in DB#4.

**Table 4:** Comparison between evaluation metrics of the GMED muscle segmentation in four databases. DC: Dice coefficient, ASD: average symmetric surface distance, AIE: average intensity error, AVE: average volume error, p: p-value of the difference between 5 and 6layers models (Student’s t-test if normal distribution, Wilcoxon signed rank test otherwise, with Bonferroni correction), n.s.: not significant.

	DC↑			ASD [mm]↓			AVE [%]↓			AIE [HU]↓		
	5layers,20	6layers,50	p	5layers,20	6layers,50	p	5layers,20	6layers,50	p	5layers,20	6layers,50	p
DB#1 (N = 50)	0.951 ± 0.015	0.961 ± 0.011	*	0.910 ± 2.436	0.432 ± 0.132	*	4.426 ± 3.303	2.355 ± 2.038	*	1.132 ± 0.906	0.811 ± 0.663	n.s
DB#2 (N = 18)	0.915 ± 0.018	0.924 ± 0.015	*	2.195 ± 3.359	1.811 ± 2.029	n.s	3.681 ± 3.971	2.869 ± 2.048	n.s	1.915 ± 1.685	0.959 ± 0.665	*
DB#3 (N = 10)	0.879 ± 0.049	0.938 ± 0.012	*	2.090 ± 0.807	0.985 ± 0.255	*	8.500 ± 9.491	3.033 ± 1.950	n..s	0.452 ± 0.324	0.603 ± 0.253	n.s
DB#4su (N = 20)	0.955 ± 0.017	0.974 ± 0.005	*	0.762 ± 0.543	0.288 ± 0.057	*	4.304 ± 6.074	1.520 ± 0.794	*	0.767 ± 0.409	0.216 ± 0.109	*
DB#4st (N = 20)	0.922 ± 0.048	0.969 ± 0.005	*	0.985 ± 0.667	0.314 ± 0.058	*	6.195 ± 7.852	1.347 ± 0.842	*	0.640 ± 0.364	0.198 ± 0.102	*

Table 5 summarizes the predictive uncertainty and its correlations with the segmentation accuracy (DC) of the GMED muscle in the four databases. With the four databases combined, both models yielded strong correlations, where the average PCCs for the *5layers,20* and *6layers,50* models were -.85 and -.60, respectively. Table 6 shows the AUROCs of failure detection at the four databases. The median AUROCs by the *6layers,50* were .963 and .995 for inaccurate (-2*σ*) and failed (-3*σ*) segmentation detection, respectively.

**Table 5:** Predictive uncertainty (mean ± standard deviation ”std”) and correlation (Pearson correlation coefficient, *ρ*) with Dice coefficient of the GMED muscle in four databases.

Model	Predictive uncertainty ↓			
	5layers,20		6layers,50	
	Mean ± std (× 10^–4^)	*ρ*	Mean ± std (× 10^–4^)	*ρ*
DB#1 (N = 50)	4.601 ± 1.458	-0.78	2.678 ± 0.593	-0.72
DB#2 (N = 18)	5.703 ± 1.703	-0.77	3.367 ± 1.367	-0.88
DB#3 (N = 10)	14.142 ± 5.294	-0.82	2.710 ± 0.523	0.12
DB#4su (N = 20)	5.225 ± 2.675	-0.93	2.603 ± 0.539	-0.88
DB#4st (N = 20)	8.774 ± 5.142	-0.97	3.102 ± 0.851	-0.88

**Table 6:** Accuracy (area under receiver operator curve (AUROC)) of the segmentation failure detection based on the predictive uncertainty and segmentation accuracy (Dice coefficient; DC) of the GMED muscle in the four databases. σ indicates the threshold computed based on the median absolute deviation of DC and used for the detection of inaccurate (-2σ) and failed (-3σ) segmentations.

Model	AUROC ↑			
	5layers,20		6layers,50	
	-2σ	-3σ	-2σ	-3σ
DB#1 (N = 50)	0.989	0.990	1.000	0.990
DB#2 (N = 18)	0.889	0.882	1.000	–
DB#3 (N = 10)	0.813	0.813	–	–
DB#4su (N = 20)	1.000	1.000	1.000	1.000
DB#4st (N = 20)	1.000	1.000	1.000	1.000
Median	0.989	0.990	1.000	1.000

### Muscle/bone assessment

Table 7 compares the volume and mean HU prediction applied to the GT and auto segmentations in DB#1 obtained from the *6layers,50* model. The measurements of the unaffected and affected hip OA sides were reported separately. Most structures exhibited substantial agreement between the GT and auto measurements on both sides (*ρ* ≥ *.*95). The PI muscle showed weak agreement in the volume and HU measurements, whereas the TFL muscle showed weak agreement only in mean HU. In both the volume and mean HU predictions, the unaffected side has shown a slightly larger MAE than the affected side. MAE of the predicted volumes at the bones and muscles for the affected and unaffected sides was 1.77±1.06 cc/m^2^ and 1.89±1.17 cc/m^2^, respectively. The MAE of the mean HU for the affected and unaffected sides was 1.46±0.95 HU and 1.38±0.89 HU, respectively. Notably, a sub-HU MAE was obtained at the GMAX, GMED, AD, BF, RF, SM, VLVI, VM muscles on the affected and unaffected sides.

**Table 7:** Comparison between affected and unaffected sides of the muscles and bones in DB#1 in terms of normalized volume and mean HU using ground truth (GT) and auto (Auto) segmented labels.

		Normalized Volume [cc/m^2^]							Mean HU [HU]						
		Affected			Unaffected				Affected			Unaffected			
Group	Structure	GT	Auto	MAE↓	GT	Auto	MAE↓	CCC↑	GT	Auto	MAE↓	GT	Auto	MAE↓	CCC↑
Hip muscles	GMAX	230.27	230.90	3.65	255.38	255.56	4.05	1.00	19.33	19.33	0.52	25.03	25.04	0.53	1.00
	GMED	94.92	95.81	2.01	104.32	105.99	2.83	0.99	29.22	29.56	0.85	34.75	35.08	0.84	1.00
	GMIN	21.72	21.98	1.70	22.52	22.32	1.53	1.69	32.74	32.86	1.67	38.11	38.05	1.54	0.99
	ILI	34.14	33.99	1.19	37.95	37.91	1.24	0.99	50.99	52.43	1.69	53.45	54.51	1.37	0.97
	OE	12.95	12.79	0.81	13.37	13.22	0.90	0.96	30.11	31.06	2.29	34.98	35.93	2.30	0.97
	OI	13.82	13.22	0.73	14.72	14.23	0.61	0.97	39.65	40.31	1.48	43.53	44.19	1.40	0.99
	PE	11.07	10.95	0.58	11.47	11.34	0.48	0.98	36.86	38.03	1.64	39.57	40.37	1.37	0.97
	PI	8.25	7.75	1.24	9.16	8.82	1.17	0.90	28.06	31.36	4.70	32.35	34.93	4.26	0.81
	PSOAS	19.35	18.56	1.43	21.96	20.93	1.67	0.97	42.08	43.46	1.55	44.03	45.36	1.50	0.98
Thigh muscles	AD	188.02	189.11	3.82	210.85	211.59	4.31	1.00	36.63	36.82	0.61	39.67	39.81	0.49	1.00
	BF	71.30	71.31	1.85	76.28	76.60	2.64	0.99	34.23	34.40	0.78	36.26	36.39	0.80	1.00
	GRAC	19.66	19.70	0.77	20.44	20.37	0.96	0.98	27.37	28.40	1.42	28.52	29.56	1.42	0.99
	RF	49.35	49.10	1.50	54.33	53.94	1.59	0.99	45.66	46.15	0.57	45.62	46.01	0.51	0.99
	SART	38.97	38.76	1.04	39.28	39.17	1.25	0.99	30.20	31.15	1.16	30.76	31.71	1.23	0.99
	SM	50.26	50.63	2.39	54.53	54.90	2.71	0.97	29.69	30.06	0.86	33.50	33.74	0.70	1.00
	ST	41.66	41.78	1.83	44.03	44.41	2.23	0.98	34.19	34.80	1.46	36.59	37.16	1.44	0.98
	TFL	19.80	19.85	0.95	19.89	19.94	0.97	0.98	23.85	24.83	1.31	25.86	27.01	1.40	0.99
	VLVI	237.39	238.48	4.50	253.34	253.95	3.82	1.00	47.84	48.12	0.65	49.93	50.17	0.59	1.00
	VM	104.64	105.33	3.06	113.61	115.50	2.96	0.98	45.65	45.55	0.63	46.67	46.58	0.61	0.99
Bones	PELVIS	122.26	122.47	0.85	121.49	121.63	0.77	1.00	320.95	321.56	1.51	334.44	335.14	1.31	1.00
	FEMUR	169.62	170.27	1.27	169.65	170.23	1.07	1.00	447.29	447.35	1.51	464.81	464.79	1.40	1.00
	SACRUM	94.22	94.00	1.74	-	-	-	0.97	190.39	192.60	3.34	-	-	-	0.99
Mean ± SD				1.77 ± 1.06			1.89 ± 1.17				1.46 ± 0.95			1.38 ± 0.89	

CCC: Concordance correlation coefficient between GT and predicted measurements, MAE: mean absolute error between GT and predicted measurements. The structures’ abbreviations are listed in Table 2.

∗ The measurements on the whole sacrum were reported since it was not separated into right/left.

**Fig. 8:**
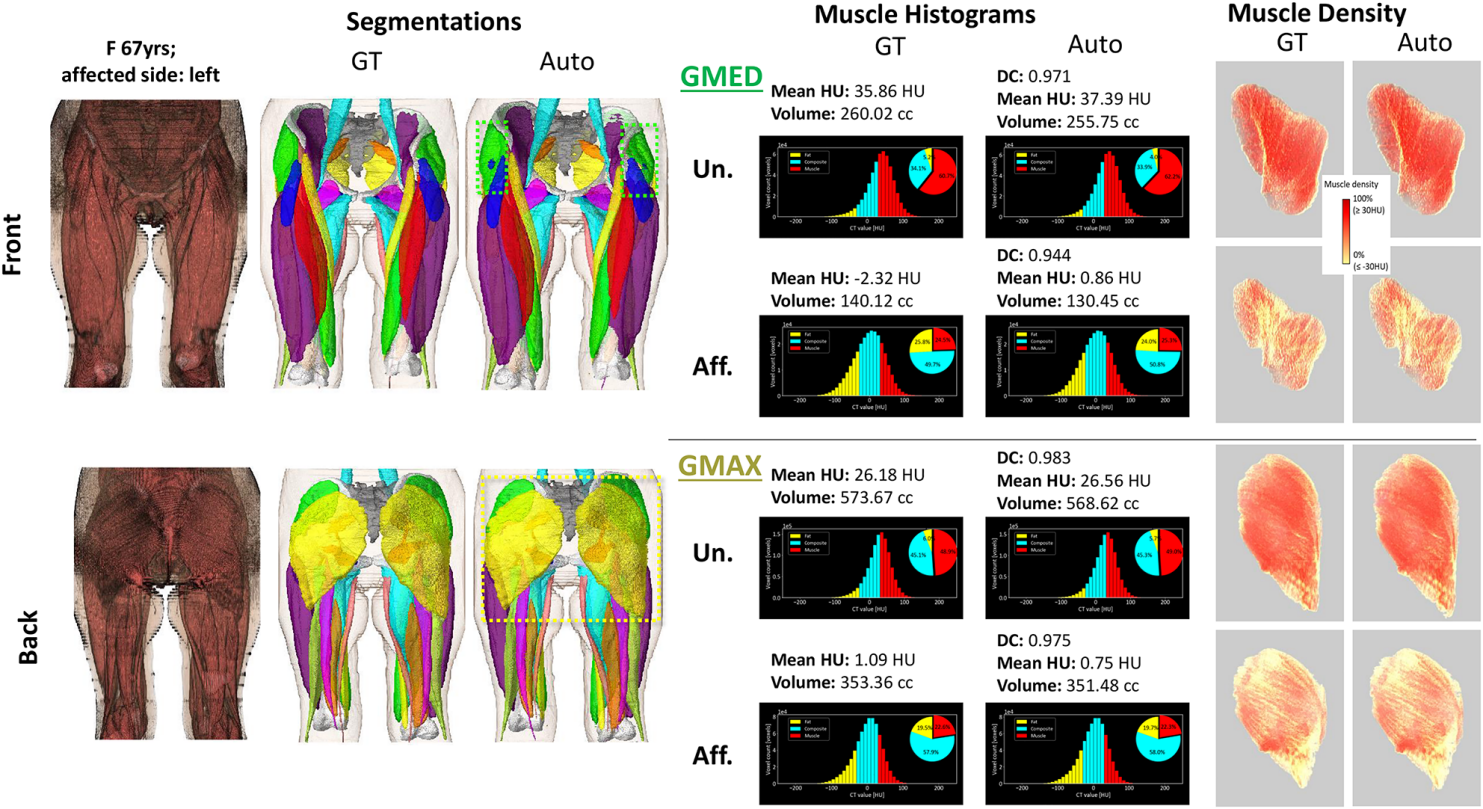
Ground-truth (GT) and predicted (Auto) segmentations of the unaffected (Un.) and affected (Aff.) sides of a representative hip OA case (median DC in Fig. 4) with diagnostic biomarkers, histograms, and muscle density visualizations of the gluteus maximus (GMAX) and gluteus medius (GMED) muscles.

Figure 8 shows representative case (median DC) segmentations with muscle histograms and 3D volume rendering of muscle density of the GMAX and GMED muscles. High reproducibility of the GT-based histograms and muscle density visualizations could be observed. In particular, the auto segmentations could comprehensively reproduce lean muscle (red) and fat (yellow) portions.

### Impact of the training and inference settings

#### Number of training images

Table 8 shows the impact of the number of training images on the segmentation accuracy (DC, ASD) of the 6layers model applied to DB#1. The highest accuracy was obtained when 40 cases were used. However, no statistically significant differences were observed in comparison to 30 cases. Compared with cases fewer than 30 cases, statistically significant differences were observed. In addition, strong correlations between DC and the predictive uncertainty were obtained in all numbers of training cases (see Supplementary Fig.A.6,b). This emphasizes the generalizability of the predictive uncertainty as a predictor of the segmentation accuracy regardless of the number of training images.

**Table 8:** Impact of the number of training cases on the segmentation accuracy of the 6-layers model. DC: Dice coefficient, ASD: Average symmetric surface distance, n.s.: not significant, *: *p* < 0.05, **: *p* < 0.01.

No. training cases	DC↑	ASD [mm]↓
10	0.931 ± 0.034	0.699 ± 0.533
	}**	}*
20	0.941 ± 0.019	0.549 ± 0.209
	}**	}*
30	0.945 ± 0.014	0.504 ± 0.174
	} n.s	} n.s
40	0.947 ± 0.013	0.488 ± 0.162

#### Number of dropout samples.

Table 9 shows the impact of the number of training samples on the segmentation accuracy by the 6layers model applied to DB#1. No improvement was observed by increasing the samples to larger than 10 samples. This indicates that 10 samples are sufficient to obtain a stable performance by the model.

**Table 9:** Impact of the number of dropout samples on the segmentation accuracy.

No. dropout samples	DC↑	ASD [mm]↓
1	0.941 ± 0.014	0.548 ± 0.210
5	0.946 ± 0.013	0.526 ± 0.189
10	0.947 ± 0.013	0.524 ± 0.185
15	0.947 ± 0.013	0.525 ± 0.186
20	0.947 ± 0.013	0.521 ± 0.183
50	0.947 ± 0.013	0.524 ± 0.185

#### Estimation method of the predictive uncertainty.

Table 10 shows the correlation between the uncertainty, estimated using entropy and MCDS-based variance, and the segmentation accuracy (DC). Both uncertainties were estimated using the 6layers model applied to DB#1. The entropy was computed from the probability maps of a single sample (deterministic setting) following the previous studies^15,33^ whereas the MCDS was computed from the variance of 10 dropout samples. The entropy produces stronger correlations with the segmentation accuracy (mean: -.71 vs. -.69); however, the usage of a single sample led to a decreased segmentation accuracy (DC: .941±.014 vs. .947±.013; as shown in Table 9).

**Table 10:** Comparison between predictive uncertainty and segmentation accuracy (Dice coefficient) using Monte Carlo Dropout Sampling (MCDS, 10 dropout samples) and entropy. ρ: Pearson correlation coefficient.

		*ρ* ↓	
Group	Structure	Entropy	MCDS (variance)
Hip muscles	GMAX	-0.81	-0.77
	GMED	-0.73	-0.72
	GMIN	-0.67	-0.46
	ILI	-0.56	-0.59
	OE	-0.33	0.07
	OI	-0.50	-0.39
	PE	-0.80	-0.78
	PI	-0.90	-0.86
	PSOAS	-0.91	-0.79
Thigh muscles	AD	-0.80	-0.83
	BF	-0.92	-0.96
	GRAC	-0.80	-0.78
	RF	-0.89	-0.95
	SART	-0.72	-0.78
	SM	-0.87	-0.90
	ST	-0.76	-0.92
	TFL	-0.57	-0.55
	VLVI	-0.73	-0.77
	VM	-0.87	-0.81
Bones	PELVIS	-0.55	-0.39
	FEMUR	-0.62	-0.61
	SACRUM	-0.34	-0.68
Mean		-0.71	-0.69

## Discussion

This study validated a DL model for the segmentation of MSK structures with uncertainty estimation in clinical CT images. The novelty of this work is that it showed the usability of the predictive uncertainty for predicting the MSK segmentation accuracy and detecting segmentation failures in databases of CT images from multi-manufacturers/scanners and with disease and positioning variations, such as supine and standing, and with different scales, including a large-database with 2,579 CTs. This showed the possibility of using the predictive uncertainty as a tool for detecting the failed segmentation in u*nannotated* CT images. The study also exhibited the potential of the *6layers,50* model in producing accurate segmentations for assessing the muscle/bone volume and mean intensity, with DC > 0.90 in almost all the muscles and > 0.95 in the bones (see Supplementary Fig. A.1). The validation on the external databases has shown high generalizability of the model’s performance, where a DC > 0.95 and an AIE < 1 HU were obtained in evaluating the GMED segmentations, and the predictive uncertainty could detect the cases with segmentation failures.

Systematic improvements were observed using the *6layers,50* model at all the structure groups, regardless of the disease status. However, the PI muscle showed the smallest DC of 0.845 ± 0.091, with the largest ASD, AIE, and AVE (see Supplementary Figs. A.1, A.2, A.4, A.5). The degraded accuracy could be interpreted by the location of this muscle among various bony, abdominal, and vascular structures, making it challenging for automated segmentation. 3D segmentation models^47^ might improve the segmentation accuracy of this muscle as they better involve the volumetric relationships with the surrounding structures.

The predictive uncertainty was investigated in several studies to predict the segmentation accuracy in medical images.^7,15,33–35^. Nowak et al. investigated the predictive uncertainty (entropy) in segmenting skeletal muscles in lumbar-level CT slices from dual centers with CT scanners from multiple manufacturers. Their study showed the applicability of the predictive uncertainty on the data from both centers; however, it was only applied to 2D CT slices, with the muscles combined into a single label. Mehtrash et al. investigated the predictive uncertainty (entropy) based on ensemble models. The method was validated on multiple structures at MRIs, and strong correlations between the predictive uncertainty and segmentation accuracy were reported. However, both studies did not address the segmentation of individual muscles or bones and did not investigate the impact of practically important factors, such as disease condition or numbers of training data on the segmentation accuracy^15,33^. In our experiments, we attempted to use the entropy of single samples and observed slightly improved correlations with the segmentation accuracy. However, the segmentation accuracy has decreased. Indeed, larger numbers of 10 samples seem to improve the overall accuracy (See Table 9). Compared with the ensemble approach^33^, the MCDS approach showed a good balance between the segmentation accuracy, computation time, and accuracy of the predictive uncertainty.

Compared with the baseline model^7^, this study showed a potential improvement when increasing the depth of the segmentation model and the number of training data. Increasing the training data to larger than 20 cases improved overall, as shown in Fig. 4. Other studies have also investigated the segmentation of thigh muscles from CT images^48–50^. However, the number of cases was smaller, making the comparison invalid. Recently, Kim et al. attempted a 3D UNETR^47^ for the segmentation of the full thigh muscles^22^. The model was trained on a larger dataset (60 cases) and tested on 12 cases; however, the dataset included only patients with hip fractures, and it showed lower accuracy (DC=0.84; ASSD=1.419±0.91 mm). These comparisons collectively emphasize the higher accuracy of the improved model and the uniqueness of our fully annotated database (DB#1) and validation of external databases (DB#2-4) regarding the number of cases and the diversity of disease, patient positioning, and imaging conditions.

The assessment of the volume and intensity of the muscles and bones are among the ultimate goals of automated MSK image segmentation. In particular, the mean HU measured at abdominal muscles has shown a higher potential to predict age-related adverse outcomes compared with the muscle area^9^. To our knowledge, this is the first study to investigate the accuracy of these measurements in automatically segmented hip and thigh MSK structures in CT images. High accuracy of the volumes and mean HU of most muscles and bones in HOA patients was obtained. Furthermore, the validation experiment on the four databases showed the robustness of the improved model in the segmentation of the GMED muscle with respect to the multi-manufacturer/scanners and disease variations. These findings indicate the potential usability of the segmentation model for hip-to-knee MSK assessments in clinical routines. The rapid inference time (∼3 min) of the entire CT volume adds to the model’s practicality for adoption in surgical planning or musculoskeletal simulation platforms. Furthermore, the muscle-wise density visualization depicted in Fig. 7 would help in the rapid and comprehensive assessment of muscle quality under several conditions, such as hip OA, cancer, sarcopenia, and obesity^23^.

On the other hand, MSK segmentation approaches in magnetic resonance images (MRIs) are attracting attention due to patient safety and high soft tissue contrast^51,52^, and the possibility of quantifying the muscle/fat composition using special sequences, such as Dixon^53^. However, MRIs usually require a long scanning time, represent various characteristics based on the acquisition sequence, and cover limited fields of view (FOVs). This necessitates integrating multiple acquisitions and registration processing to assess the whole knee-to-hip^52^, which could be limited to a few research-purposed databases^25^.

This study has the following limitations. The 2D segmentation model, even though it has a rapid inference time, does not capture the 3D information of neighboring structures, which affects the segmentation of small structures, such as the PI muscle. State-of-the-art 3D models, such as nnUNet^54^ or swin UNETR^55^, may potentially improve the segmentation of the small muscles; however, those models are known for their higher computational cost and longer inference time than their 2D counterparts^56^. This study did not investigate those models because it aimed to explore the potential improvements in the baseline 2D model and its predictive uncertainty, leveraging its fast inference time for large-scale analysis. Nevertheless, future studies should investigate the potential of 3D models to improve the segmentation accuracy of small structures. Furthermore, the small hip muscles (OI and OE muscles) showed low AUROC in failure detection based on the predictive uncertainty. The usage of the auto segmentations of those muscles requires attention in *unannotated* databases.

The failure detection approach and improved model (*6layers,50*) create a basis for several future directions in our research. The model’s potential in analyzing the disease progression of individual bones and muscles in large-scale databases of *unannotated* CTs will be investigated. Cases with segmentation failures could be detected based on the predictive uncertainty and excluded or refined by human annotators for downstream MSK analyses. Furthermore, the extension of the segmentation model to predict the MSK structures in other regions, such as the abdomen and back muscles, is currently under development. Furthermore, a few muscles in the hip, such as the quadratus femoris and Gemelli muscles, were not addressed, besides combining several muscles, such as the adductors, into a single label due to the challenging boundary definition. These structures will be addressed in our future work by involving higher-resolution images, such as from photon-counting CTs. This study focused on hip OA as a target MSK pathology. As the model has shown possible dependency on the disease severity in the large-scale analysis, we plan to further investigate its performance on other muscle pathologies, such as muscular dystrophy^13^, cachexia^14^, and sarcopenia^15^.

## Conclusions

This study validated a DL model for MSK segmentation with uncertainty estimation in clinical CT images. The improved model (*6layers,50*) allowed for the automated, rapid, and accurate assessment of the volume and density of the hip and thigh bones and muscles from clinical CT images. The study has shown an impact of the disease severity on the model’s performance, and the usability of the predictive uncertainty as a tool for predicting the segmentation accuracy and failure detection in individual MSK structures at *unannotated* CT image databases. The high segmentation and muscle volume/density estimation accuracy, along with the high accuracy in failure detection, exhibited the model’s reliability for the analysis of individual MSK structures in large-scale CT databases.

## Supplementary Information

Supplementary Information.

## Data Availability

The datasets and the pre-trained models used and analyzed in the current study are available from the corresponding author upon reasonable request.
