# Supplementary material for: Validation of musculoskeletal segmentation model with uncertainty estimation for bone and muscle assessment in hip-to-knee clinical CT images
Source: Sci Rep. 2025 Jan 2;15:125. doi: 10.1038/s41598-024-83793-7 (PMC11696574; doi:10.1038/s41598-024-83793-7)
Supplement: Supplementary file 1 — Supplementary Information. [file 41598_2024_83793_MOESM1_ESM.pdf]

Validation of musculoskeletal segmentation model with uncertainty estimation for bone and muscle assessment in hip-to-knee clinical CT images

Mazen Soufi[1,*], Yoshito Otake[1,*], Makoto Iwasa[2], Keisuke Uemura[2], Tomoki Hakotani[1], Masahiro Hashimoto[3], Yoshitake Yamada[3], Minoru Yamada[3], Yoichi Yokoyama[3], Masahiro Jinzaki[3], Suzushi Kusano[4], Masaki Takao[5], Seiji Okada[2], Nobuhiko Sugano[6], and Yoshinobu Sato[1,*]

[1]Division of Information Science, Graduate School of Science and Technology, Nara Institute of Science and Technology, 8916-5 Takayama-cho, Ikoma, Nara 630-0192, Japan
[2]Department of Orthopedic Surgery, Graduate School of Medicine, Osaka University, 2-2 Yamadaoka, Suita, Osaka 565-0871, Japan
[3]Department of Radiology, Keio University School of Medicine, 35 Shinanomachi, Shinjuku-ku, Tokyo 160-8582, Japan
[4]Hitachi Health Care Center, Hitachi Ltd., 4-3-16 Ose, Hitachi 307-0076, Japan
[5]Department of Bone and Joint Surgery, Graduate School of Medicine, Ehime University, Shitsukawa, Toon, Ehime 791-0295, Japan
[6]Department of Orthopaedic Medical Engineering, Graduate School of Medicine, Osaka University, 2-2 Yamadaoka, Suita, Osaka 565-0871, Japan
[1]msoufi,otake,yoshi@is.naist.jp

Appendices

A Muscle-wise evaluation metrics

Figures A.1-A.5 summarize the evaluation metrics and predictive uncertainty of each hip-to-knee structure (3 bones and 19 muscles) using the three models from the 5-fold cross-validation experiments. The box plots include the detailed metric values of the box plots depicted in Fig. 4.

- Fig.A.1: Dice coefficient (DC), corresponding to Fig. 4(a).
- Fig.A.2: Average symmetric surface distance (ASD, mm), corresponding to Fig. 4(a).
- Fig.A.3: Predictive uncertainty, corresponding to Fig. 4(b).
- Fig.A.4: Average volume error (AVE,%), corresponding to Fig. 4(c).
- Fig.A.5: Average intensity error (AIE,HU), corresponding to Fig. 4(c).

B Relationship between predictive uncertainty and segmentation accuracy (Dice coefficient).

Figure A.6 depicts the relationship between the predictive uncertainty and segmentation accuracy (DC) in terms of a) the segmentation model and b) the number of training images. The two plots correspond to the box plots in Fig. 4 and Table 8, respectively. Each point represents a single muscle/bone (right and left sides combined). All experiments were performed at DB#1.

C Representative segmentation results from the four databases used in the study

Figure A.7 and A.8 shows segmentation results from the databases DB#1-4 used in this study, with qualitative and quantitative evaluations of the GMED and other structures. In each case, the models *5layers,20* and *6layers,50* predicted the upper and lower segmentations, respectively. For each database, the upper and lower cases correspond with the 5th (▲) and 95th (▼)

quantiles of the predictive uncertainty visualized in Fig. 6. The cases with lower uncertainty had higher segmentation accuracy in both models and vice versa.

D Usability of the relationship between predictive uncertainty and segmentation accuracy for detection of inaccurate and failed segmentations.

Figures A.9 and A.10 show the relationships between the predictive uncertainty and segmentation accuracy for detecting inaccurate and failed segmentations.

- Fig.A.9: Each structure in DB#1.
- Fig.A.10: GMED in the four databases DB#1-4.

E Usability of the predictive uncertainty for detecting inaccurate and failed segmentations in large-scale databases.

Figure A.11 shows the usability of the predictive uncertainty in detecting inaccurate and failed segmentations in a large-scale database consisting of 2,579 CT images. The scatter plot shows the average predictive uncertainty of all structures by the *5layers,20* and *6layers,50* models. The right side depicts representative cases selected based on the thresholds shown in Fig. 5. Most cases have shown lower predictive uncertainty by the *6layers,50* model. The detected segmentation failure case (⋆) had a large variation in the hip positioning, possibly caused by the patient's discomfort due to the disease.

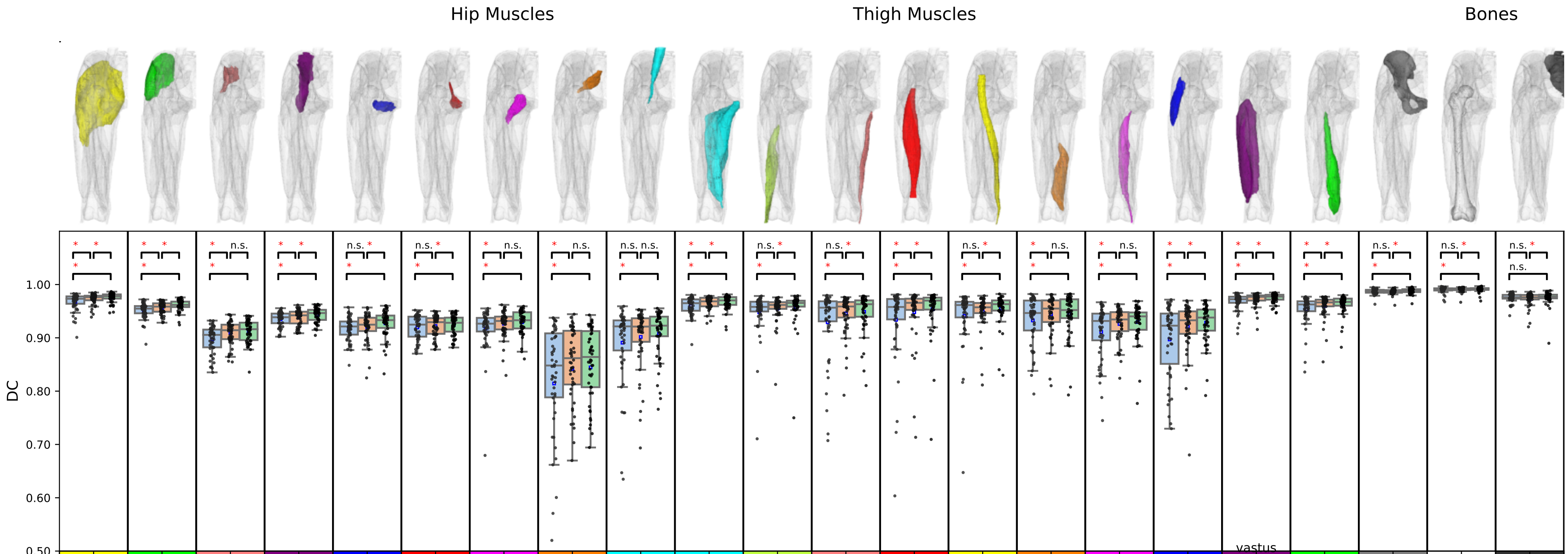

	gluteus maximus	gluteus medius	gluteus minimus	iliacus	obturator externus	obturator internus	pectineus	piriformis	psoas major	adductors	biceps femoris	gracilis	rectus femoris	sartorius	semi membranosus	semi tendinosus	tensor fasciae latae	vastus lateralis and vastus intermedius	vastus medialis	pelvis	femur	sacrum
5layers,20 (conv.)	0.968 ±0.016	0.951 ±0.015	0.897 ±0.026	0.935 ±0.014	0.917 ±0.022	0.920 ±0.023	0.918 ±0.041	0.814 ±0.144	0.891 ±0.071	0.960 ±0.017	0.948 ±0.042	0.929 ±0.067	0.934 ±0.072	0.941 ±0.055	0.933 ±0.046	0.911 ±0.051	0.897 ±0.068	0.968 ±0.016	0.954 ±0.028	0.987 ±0.005	0.990 ±0.005	**0.975 ±0.009**
5layers,50	0.973 ±0.010	0.956 ±0.011	0.909 ±0.023	0.938 ±0.015	0.920 ±0.026	0.922 ±0.020	0.926 ±0.025	0.842 ±0.100	0.902 ±0.057	0.965 ±0.011	0.955 ±0.026	0.946 ±0.037	0.948 ±0.052	0.950 ±0.032	**0.944 ±0.037**	0.926 ±0.031	0.921 ±0.048	0.973 ±0.012	0.960 ±0.022	0.986 ±0.005	**0.991 ±0.004**	0.973 ±0.013
6layers,50	**0.975 ±0.009**	**0.961 ±0.011**	**0.912 ±0.021**	**0.943 ±0.014**	**0.927 ±0.025**	**0.925 ±0.019**	**0.929 ±0.022**	**0.845 ±0.091**	**0.908 ±0.046**	**0.967 ±0.013**	**0.957 ±0.033**	**0.949 ±0.036**	**0.954 ±0.045**	**0.955 ±0.029**	**0.944 ±0.040**	**0.930 ±0.034**	**0.929 ±0.036**	**0.975 ±0.009**	**0.963 ±0.018**	**0.988 ±0.005**	**0.991 ±0.005**	**0.975 ±0.014**

Figure A.1. Dice Coefficient (DC) in DB#1(N=50), corresponds to Fig. 4(a).

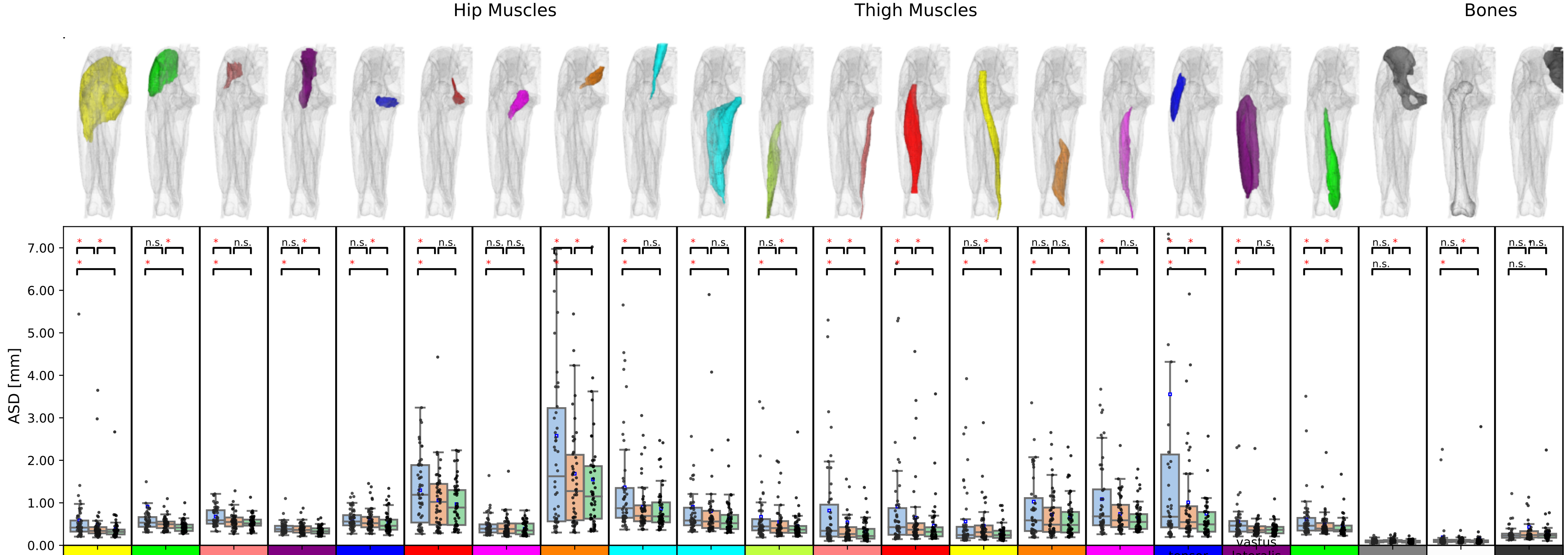

	gluteus maximus	gluteus medius	gluteus minimus	iliacus	obturator externus	obturator internus	pectineus	piriformis	psoas major	adductors	biceps femoris
5layers,20 (conv.)	0.588 ±0.748	0.910 ±2.436	0.676 ±0.221	0.412 ±0.141	0.623 ±0.223	1.296 ±0.804	0.454 ±0.243	2.577 ±3.006	1.364 ±1.207	0.907 ±1.165	0.670 ±0.654
5layers,50	0.482 ±0.600	0.505 ±0.158	0.569 ±0.194	0.392 ±0.130	0.599 ±0.267	1.062 ±0.728	0.437 ±0.252	1.684 ±1.580	0.903 ±0.580	0.807 ±0.948	0.549 ±0.416
6layers,50	**0.390 ±0.354**	**0.432 ±0.132**	**0.537 ±0.154**	**0.348 ±0.096**	**0.532 ±0.241**	**0.970 ±0.535**	**0.389 ±0.156**	**1.549 ±1.500**	**0.861 ±0.518**	**0.633 ±0.399**	**0.451 ±0.368**

	gracilis	rectus femoris	sartorius	semi membranosus	semi tendinosus	tensor fasciae latae	vastus lateralis and vastus [illegible]	vastus medialis	pelvis	femur	sacrum
5layers,20 (conv.)	0.818 ±1.131	0.914 ±1.348	0.560 ±0.785	1.029 ±1.374	1.086 ±0.918	3.554 ±6.729	0.558 ±0.424	0.610 ±0.565	0.097 ±0.039	0.190 ±0.405	**0.267 ±0.178**
5layers,50	0.542 ±1.370	0.651 ±0.880	0.460 ±0.472	0.723 ±0.573	0.739 ±0.423	1.011 ±1.137	0.426 ±0.292	0.490 ±0.313	0.113 ±0.053	**0.113 ±0.052**	0.431 ±0.991
6layers,50	**0.322 ±0.254**	**0.462 ±0.547**	**0.290 ±0.188**	**0.659 ±0.502**	**0.642 ±0.343**	**0.743 ±1.125**	**0.382 ±0.150**	**0.431 ±0.237**	**0.091 ±0.037**	0.152 ±0.384	0.292 ±0.309

Figure A.2. Average symmetric surface distance (ASD) in DB#1(N=50), corresponds to Fig. 4(a).

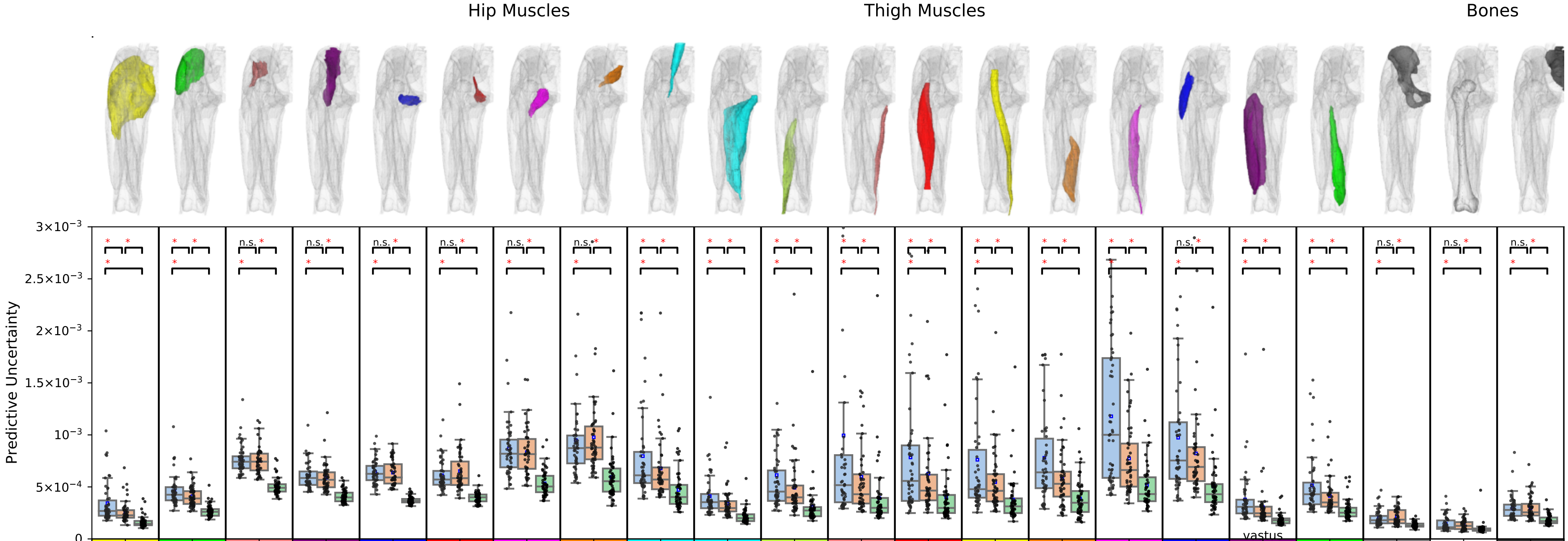

	gluteus maximus	gluteus medius	gluteus minimus	iliacus	obturator externus	obturator internus	pectineus	piriformis	psoas major	adductors	biceps femoris	gracilis	rectus femoris	sartorius	semi membranosus	semi tendinosus	tensor fasciae latae	vastus lateralis and vastus	vastus medialis	pelvis	femur	sacrum
5layers,20 (conv.)	3.4e-04 ±1.9e-04	4.6e-04 ±1.5e-04	7.6e-04 ±1.4e-04	6.1e-04 ±1.3e-04	6.4e-04 ±1.2e-04	6.1e-04 ±1.4e-04	8.7e-04 ±3.0e-04	9.5e-04 ±4.3e-04	8.0e-04 ±4.5e-04	4.1e-04 ±2.1e-04	6.1e-04 ±5.8e-04	9.9e-04 ±1.1e-03	7.8e-04 ±6.0e-04	7.6e-04 ±6.7e-04	7.8e-04 ±4.4e-04	1.2e-03 ±6.7e-04	9.7e-04 ±5.7e-04	3.8e-04 ±2.6e-04	5.1e-04 ±2.9e-04	2.0e-04 ±7.6e-05	1.5e-04 ±7.3e-05	2.9e-04 ±1.2e-04
5layers,50	2.6e-04 ±1.0e-04	4.1e-04 ±9.9e-05	7.5e-04 ±1.3e-04	5.9e-04 ±1.4e-04	6.4e-04 ±1.2e-04	6.5e-04 ±2.1e-04	8.3e-04 ±2.3e-04	9.8e-04 ±3.8e-04	6.8e-04 ±3.3e-04	3.4e-04 ±1.4e-04	4.9e-04 ±3.3e-04	6.0e-04 ±4.7e-04	6.3e-04 ±4.8e-04	5.5e-04 ±3.0e-04	5.8e-04 ±3.0e-04	7.7e-04 ±3.6e-04	8.2e-04 ±4.7e-04	3.1e-04 ±2.4e-04	4.1e-04 ±1.7e-04	2.2e-04 ±7.7e-05	1.3e-04 ±5.2e-05	3.0e-04 ±1.1e-04
6layers,50	**1.6e-04 ±5.6e-05**	**2.7e-04 ±6.0e-05**	**5.1e-04 ±8.0e-05**	**4.1e-04 ±6.0e-05**	**3.7e-04 ±3.6e-05**	**4.0e-04 ±5.4e-05**	**5.3e-04 ±1.3e-04**	**6.0e-04 ±2.2e-04**	**4.7e-04 ±2.0e-04**	**2.2e-04 ±7.2e-05**	**3.1e-04 ±2.1e-04**	**3.9e-04 ±3.3e-04**	**4.0e-04 ±2.9e-04**	**3.8e-04 ±2.4e-04**	**3.9e-04 ±1.9e-04**	**5.2e-04 ±2.5e-04**	**5.1e-04 ±3.1e-04**	**1.9e-04 ±5.6e-05**	**2.8e-04 ±9.8e-05**	**1.3e-04 ±2.6e-05**	**9.7e-05 ±5.6e-05**	**1.8e-04 ±4.9e-05**

Figure A.3. Predictive uncertainty in DB#1(N=50), corresponds to Fig. 4(b).

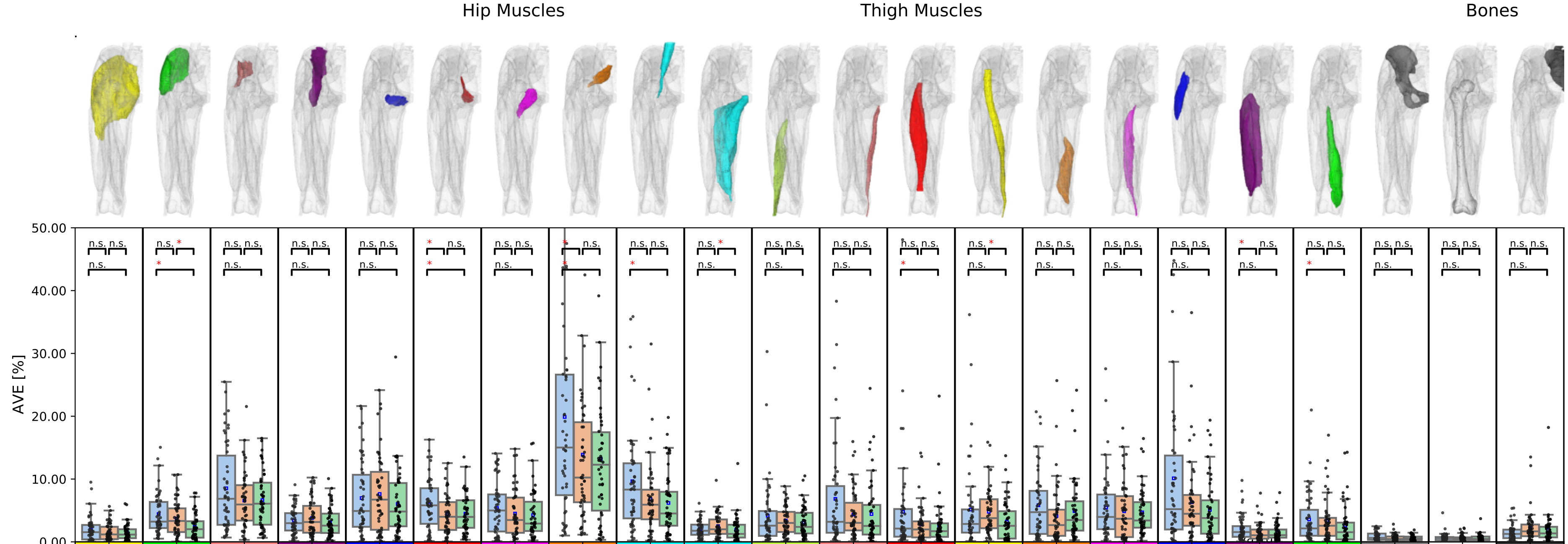

	gluteus maximus	gluteus medius	gluteus minimus	iliacus	obturator externus	obturator internus	pectineus	piriformis	psoas major	adductors	biceps femoris	gracilis	rectus femoris	sartorius	semi membranosus	semi tendinosus	tensor fasciae latae	lateralis and vastus	vastus medialis	pelvis	femur	sacrum
5layers,20 (conv.)	1.994 ±1.988	4.426 ±3.303	8.532 ±6.854	3.403 ±2.168	6.970 ±5.963	5.963 ±4.105	5.395 ±4.278	19.884 ±17.942	9.653 ±8.495	2.021 ±1.350	4.156 ±5.376	6.842 ±8.389	4.778 ±7.958	5.145 ±6.968	5.831 ±5.336	5.473 ±5.435	10.121 ±12.159	2.145 ±2.063	3.572 ±3.956	0.830 ±0.692	0.669 ±0.693	**1.493 ±1.248**
5layers,50	1.520 ±1.352	3.727 ±2.673	**6.575 ±4.480**	3.791 ±2.849	7.621 ±6.196	**4.441 ±3.097**	4.511 ±3.826	13.883 ±12.800	6.831 ±5.994	2.512 ±1.855	3.401 ±2.244	**4.362 ±3.812**	2.788 ±2.933	4.791 ±3.600	**4.109 ±4.814**	4.868 ±4.466	6.315 ±6.727	**1.532 ±1.538**	3.372 ±3.624	**0.592 ±0.551**	**0.606 ±0.534**	2.274 ±2.530
6layers,50	**1.483 ±1.304**	**2.392 ±2.037**	6.644 ±4.298	**3.139 ±2.342**	**6.022 ±5.228**	4.501 ±3.040	**4.267 ±3.794**	**13.222 ±11.905**	**6.195 ±4.896**	**1.903 ±2.014**	**3.064 ±2.409**	4.426 ±5.121	**2.622 ±3.677**	**3.236 ±3.156**	4.886 ±5.005	**4.706 ±3.664**	**5.089 ±4.973**	1.551 ±1.544	**2.593 ±3.257**	0.605 ±0.438	**0.606 ±0.603**	1.913 ±2.587

Figure A.4. Average volume error (AVE) in DB#1(N=50), corresponds to Fig. 4(c).

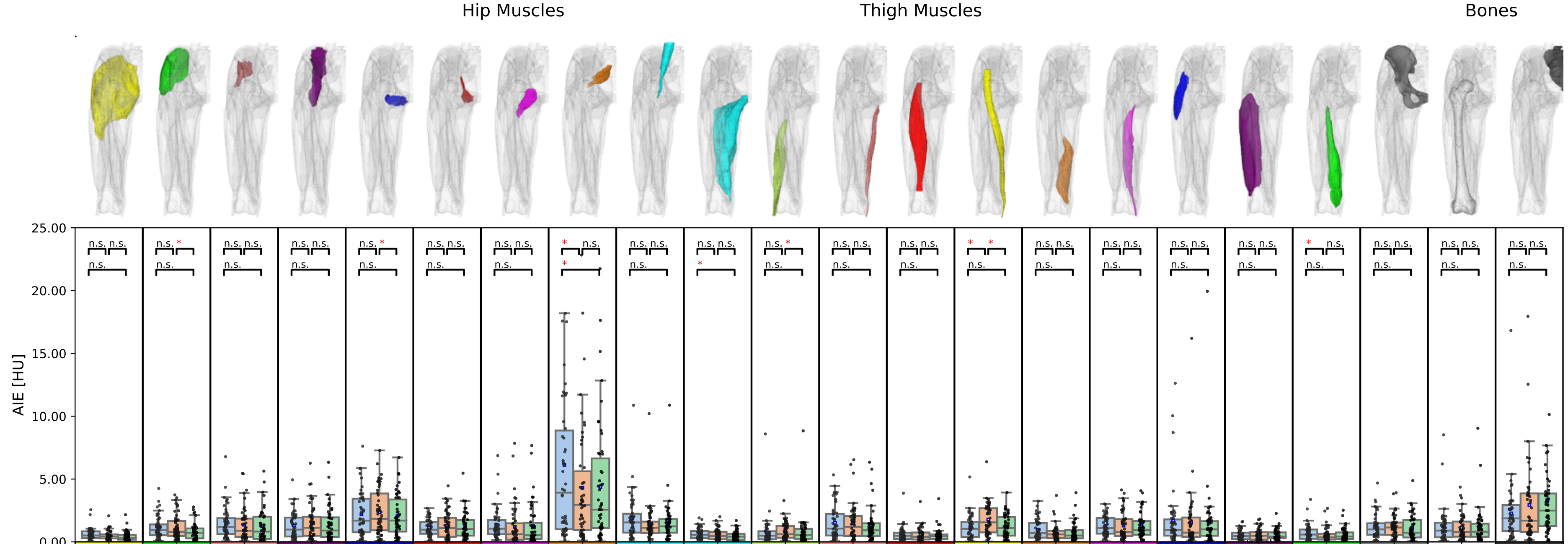

	gluteus maximus	gluteus medius	gluteus minimus	iliacus	obturator externus	obturator internus	pectineus	piriformis	psoas major	adductors	biceps femoris	gracilis	rectus femoris	sartorius	semi membranosus	semi tendinosus	tensor fasciae latae	lateralis and vastus	vastus medialis	pelvis	femur	sacrum
5layers,20 (conv.)	0.590 ±0.483	1.132 ±0.906	1.490 ±1.259	**1.306 ±1.078**	2.196 ±1.792	**1.137 ±0.685**	1.371 ±1.274	6.130 ±7.426	1.854 ±1.813	0.635 ±0.457	**0.754 ±1.240**	1.513 ±1.357	0.589 ±0.602	**1.139 ±0.950**	0.970 ±0.815	1.279 ±0.799	1.674 ±2.426	**0.524 ±0.406**	0.718 ±0.644	1.199 ±0.990	1.226 ±1.446	**2.269 ±2.506**
5layers,50	0.494 ±0.381	1.152 ±0.983	1.360 ±1.297	1.481 ±1.348	2.359 ±1.852	1.294 ±1.061	**1.190 ±1.651**	**4.300 ±4.790**	**1.398 ±1.457**	0.572 ±0.453	0.862 ±0.681	1.506 ±1.451	0.568 ±0.556	1.774 ±1.209	**0.695 ±0.655**	1.295 ±1.106	1.559 ±2.407	0.573 ±0.462	**0.516 ±0.554**	1.212 ±0.871	**1.114 ±1.001**	2.928 ±3.274
6layers,50	**0.417 ±0.393**	**0.803 ±0.669**	**1.240 ±1.276**	1.381 ±1.352	**2.056 ±1.632**	1.243 ±1.042	1.251 ±1.675	4.376 ±4.806	1.509 ±1.637	**0.470 ±0.373**	0.794 ±1.255	**1.270 ±1.359**	**0.517 ±0.541**	1.206 ±0.923	0.763 ±0.751	**1.258 ±1.012**	**1.529 ±2.812**	0.565 ±0.415	0.558 ±0.514	**1.107 ±1.002**	1.190 ±1.496	3.337 ±4.124

Figure A.5. Average intensity error (AIE) in DB#1(N=50), corresponds to Fig. 4(c).

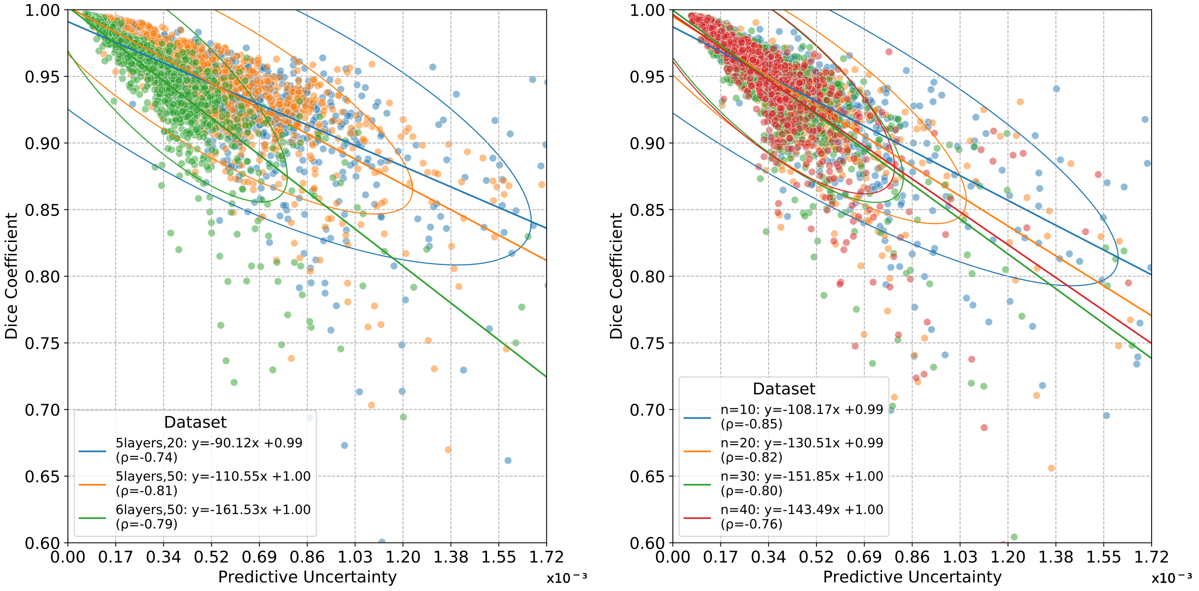

(a) Impact of segmentation models

(b) Impact of number of training cases

Figure A.6. Relationship between predictive variance (uncertainty) and segmentation accuracy (Dice coefficient) in DB#1(N=50) in terms of a) the segmentation model, and b) the number of training images. The two plots correspond to the box plots in Fig. 4 and Table 8, respectively. Each point represents a single muscle/bone (right and left sides combined). ρ indicates Pearson's correlation coefficient. Strong correlations were obtained between DC and the predictive uncertainty in all experiments.

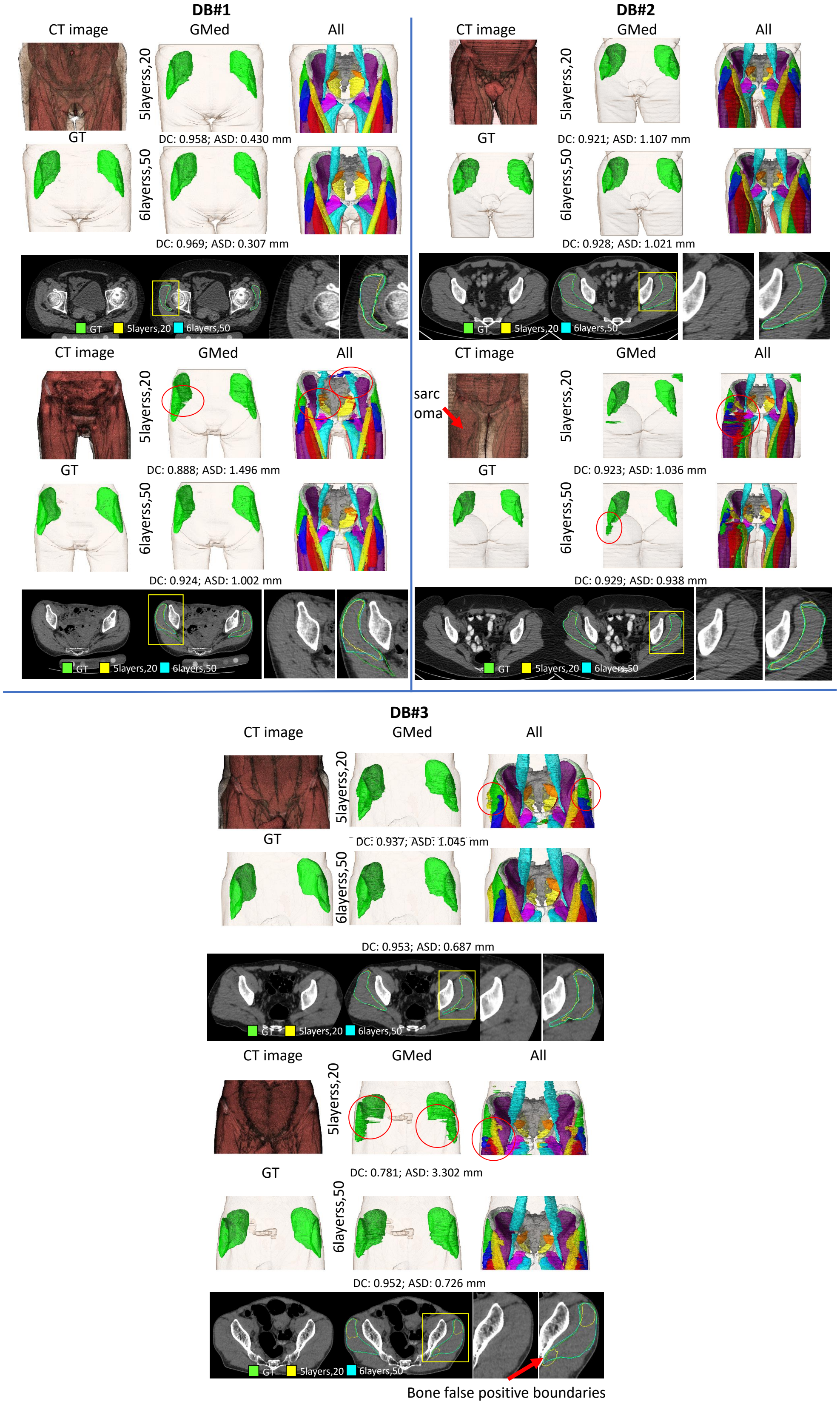

Figure A.7. Representative segmentation results from the validation databases DBs#1-3(N=50, 18 and 10, respectively), whose quantitative results are visualized in Fig. 7. For each database, the upper and lower cases correspond with the 5th (▲) and 95th (▼) quantiles of the predictive uncertainty. Red circles indicate improved locations using the 6layer,50 model. DC: Dice coefficient, ASD: Average symmetric surface distance.

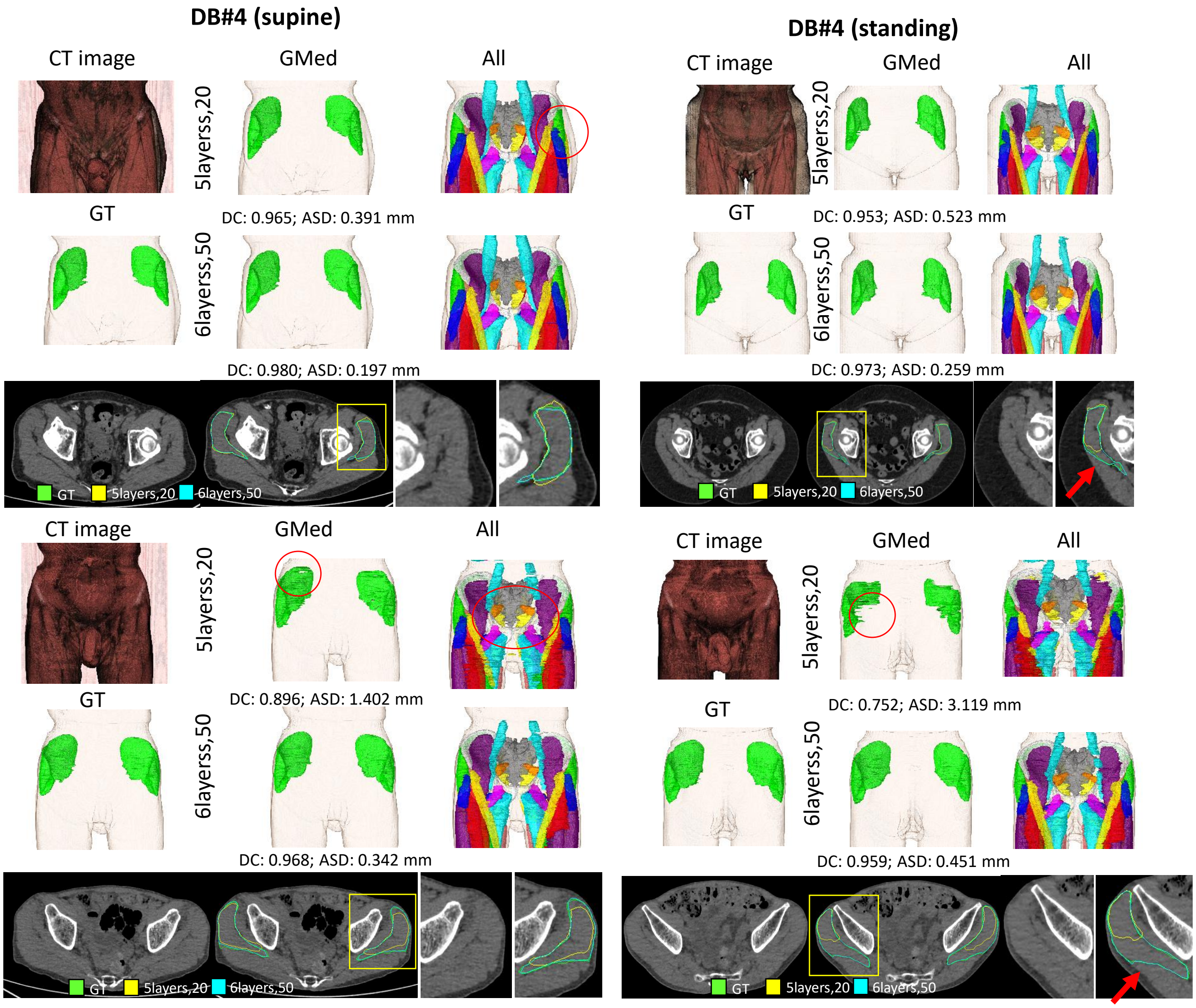

Figure A.8. Representative segmentation results from the validation database DB#4 (N=20) for the patient in supine and standing positionings, whose quantitative results are visualized in Fig. 7. For each database, the upper and lower cases correspond with the 5th (▲) and 95th (▼) quantiles of the predictive uncertainty. Red circles indicate improved locations using the 6layer,50 model. DC: Dice coefficient, ASD: Average symmetric surface distance.

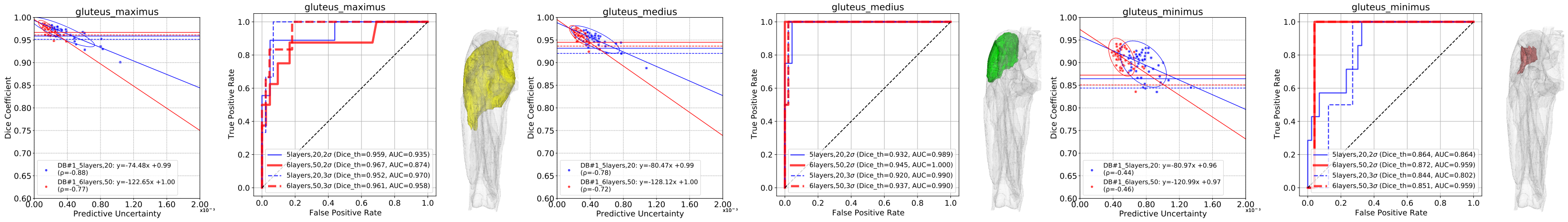

(a) Gluteus maximus (b) Gluteus medius (c) Gluteus minimus

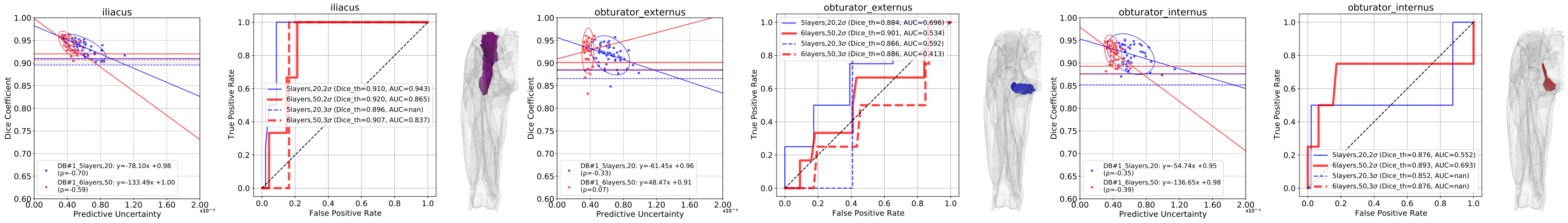

(d) Iliacus (e) Obtuator exturnus (f) Obtuator internus

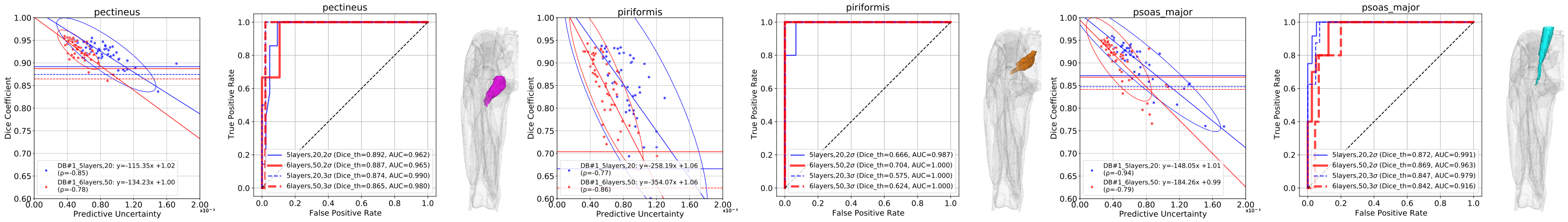

(g) Pectenius (h) Piriformis (i) Psoas major

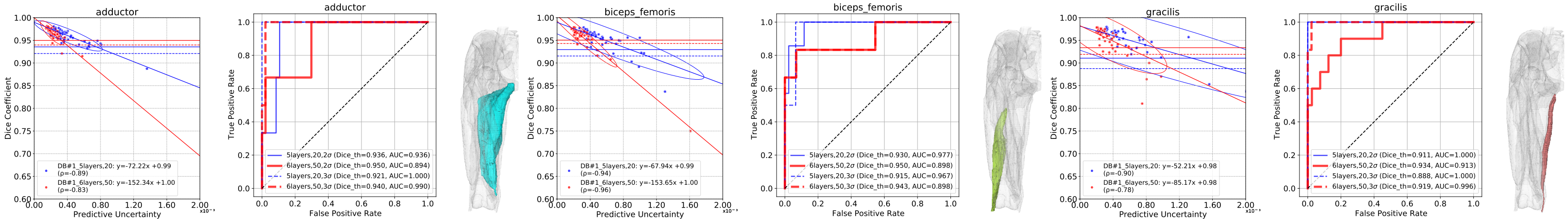

(j) Adductors (k) Biceps femoris (l) Gracilis

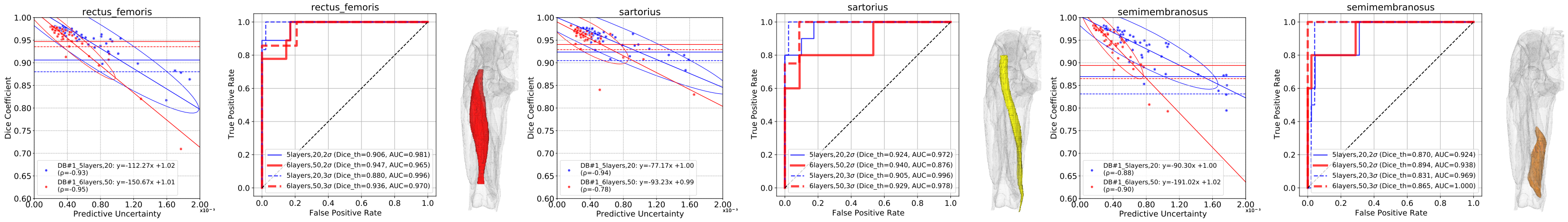

(m) Rectus femoris (n) Sartorius (o) Semimembranosus

contd.

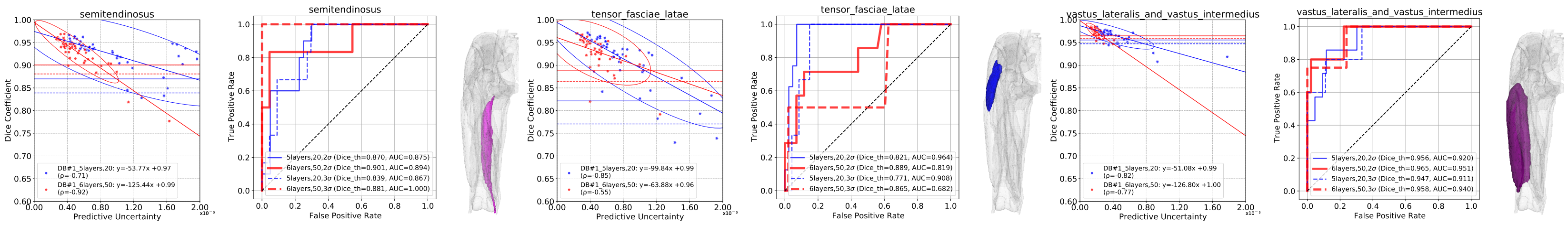

(p) Semitendinosus (q) Tensor fascia latae (r) Vastus Lateralis and Vastus Intermedius

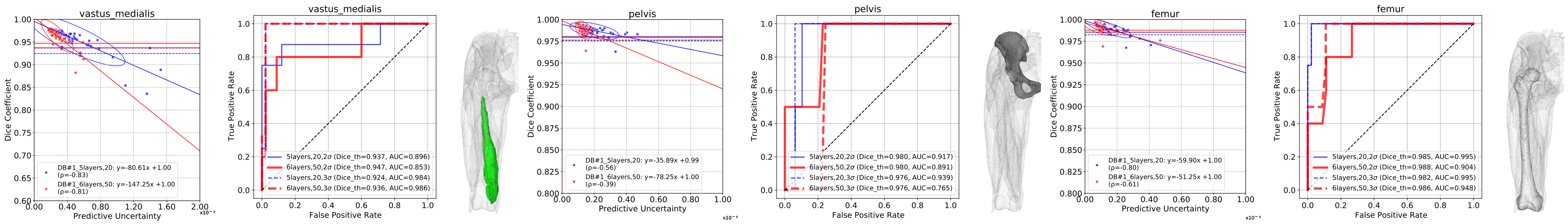

(s) Vastus medialis (t) Pelvis (u) Femur

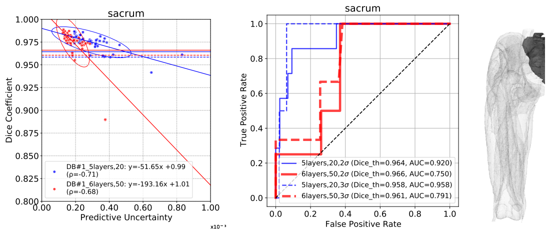

(v) Sacrum

Figure A.9. Relationship between the predictive uncertainty and segmentation accuracy (Dice coefficient; DC) in each structure at DB#1 (N=50), with receiver operating characteristic (ROC) curves of detecting the inaccurate (2σ of DC) and failed (3σ of DC) based on the predictive uncertainty. In each plot, solid and dashed lines indicate the 2σ and 3σ, respectively. Blue and red lines indicate the 5layers,20 and 6layers,50 models, respectively.

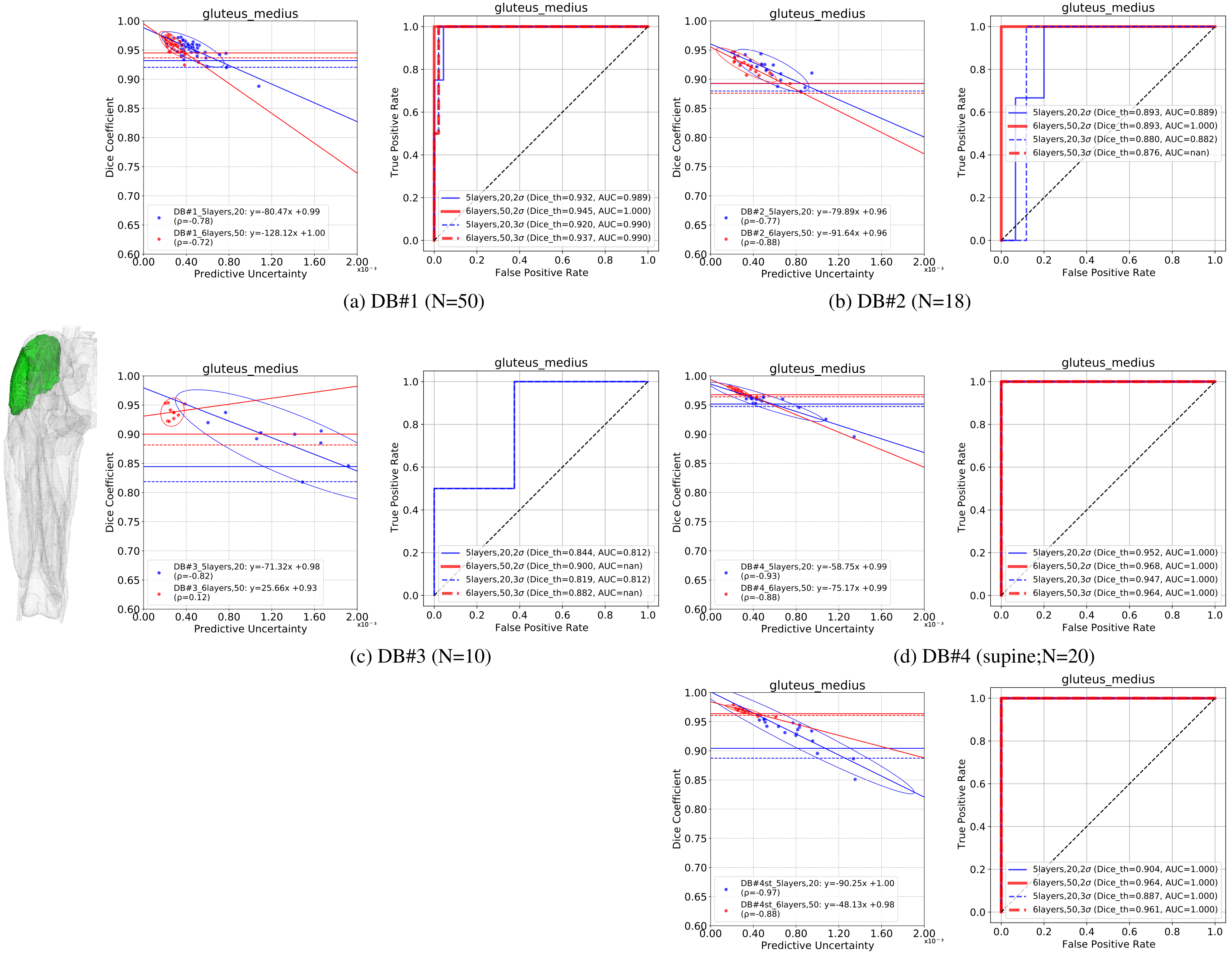

(a) DB#1 (N=50)

(b) DB#2 (N=18)

(c) DB#3 (N=10)

(d) DB#4 (supine;N=20)

(e) DB#4 (standing;N=20)

Figure A.10. Relationship between the predictive uncertainty and segmentation accuracy (Dice coefficient; DC) of the gluteus medius at the four databases, with receiver operating characteristic (ROC) curves of detecting the inaccurate (-2σ of DC) and failed (-3σ of DC). In each plot, solid and dashed lines indicate the -2σ and -3σ, respectively. Blue and red lines indicate the *5layers,20* and *6layers,50* models, respectively.

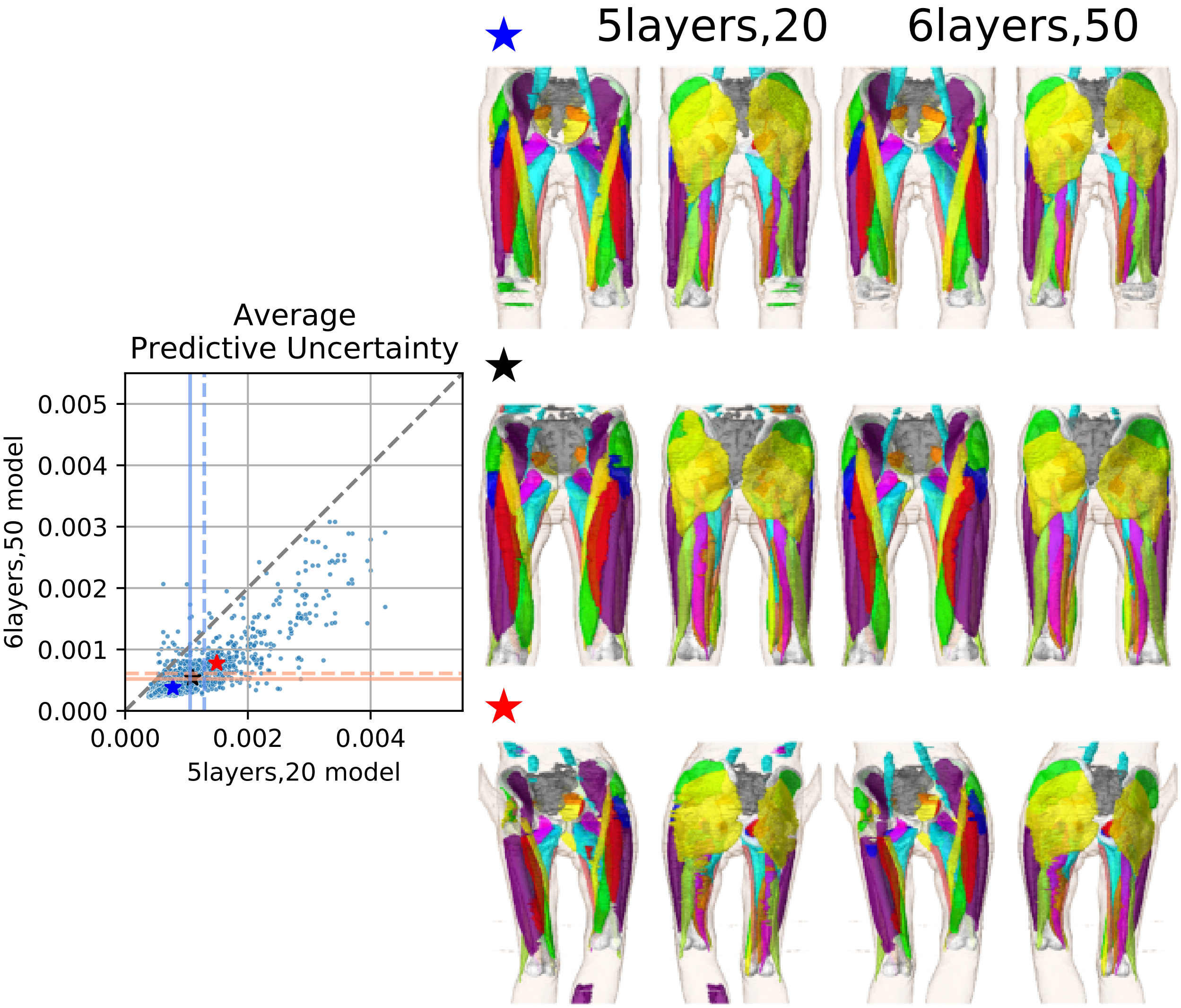

Figure A.11. Usability of the predictive uncertainty in detecting inaccurate and failed segmentations in DB#5 (N=2,579). The blue and orange lines correspond with the *5layers,20* and *6layers,50* thresholds, respectively. The solid and dashed lines correspond with the 2σ and 3σ thresholds, respectively, derived from DB#1 (See Fig. 5). The stars correspond with the representative cases based on the predictive uncertainty thresholds. ★ : $x<2\sigma$,★ : $2\sigma<x<3\sigma$ ★ : $x>3\sigma$
